# Chromosome-Scale Atlas of *Ixodes scapularis* Serine Protease Inhibitors

**DOI:** 10.3390/genes17040361

**Published:** 2026-03-24

**Authors:** Alex S. Kiarie Gaithuma, Thu-Thuy Nguyen, Albert Mulenga

**Affiliations:** College of Veterinary Medicine & Biomedical Sciences, Texas A&M University, 4474 TAMU, College Station, TX 77843-4474, USA; akiariegaithuma@cvm.tamu.edu (A.S.K.G.); ttnguyen@cvm.tamu.edu (T.-T.N.)

**Keywords:** chromosome, *Ixodes scapularis*, RNA sequencing, serine protease inhibitors, serpins, tick feeding

## Abstract

**Background/Objectives**: Ticks evade host hemostasis and immunity in part by injecting serine protease inhibitors (serpins) into the host during feeding, yet the genomic organization of tick serpins has remained unresolved. To understand how ticks deploy these proteins, there is a need to elucidate their gene structure, arrangement and copy number in the genome. **Methods**: We annotated the recent *Ixodes scapularis* chromosome-level assembly and identified all the serpin genes to build a genome-wide atlas of serpin loci identifying the gene structure and duplication patterns. The gene expression of serpins during blood meal was also analyzed. **Results**: We identified 74 serpin genes across eight chromosomes and one unplaced scaffold, with a strongly non-random distribution dominated by chromosome 10, which harbored 67.6% of serpin genes in dense tandem clusters. Most genes were intronless and encoded secreted, N-glycosylated proteins, whereas a minority were conserved two-exon loci sharing a common splice junction. Pairwise amino acid comparisons revealed exact duplicates as well as very recent and divergent paralogs, indicating continued local duplication and diversification. Expression analysis across tissues and feeding time showed that serpin expression is structured primarily by organ and feeding stage, including a late feeding increase in midgut serpins that are predicted to inhibit trypsin-like proteases. **Conclusions**: This atlas provides a comprehensive description of *I. scapularis* serpins, provides a framework for understanding tick gene structure and function, prioritizes serpins as target candidates for tick control, and functions as a library for other serpin uses in medicine and industry.

## 1. Introduction

Ticks are obligate hematophagous ectoparasites that have evolved one of the most remarkable host–parasite interfaces among arthropod vectors of disease. Unlike mosquitoes that feed only for short durations, ticks feed for long periods and remain stuck on the vertebrate host for several days up to over a week, depending on life stage and species [1]. The prolonged blood feeding exposes ticks to the whole spectrum of host defenses that comprise innate and adaptive immunity, such as complement activation, inflammation, blood clotting, platelet aggregation, vasoconstriction, and others [2,3]. To counteract the host responses, ticks secrete into the feeding site a dynamic mixture of salivary proteins that cumulatively comprises one of the most complex pharmacological repertoires in the animal kingdom [4]. This salivary cocktail evolves continuously during the course of feeding with stage-specific and time-dependent changes in protein composition that fine-tune host immune modulation [5,6].

Most of the innate immune defenses that ticks must evade to feed and transmit disease agents are serine protease-mediated pathways that are under tight regulation by serpins [7]. In humans, classical examples include α1-antitrypsin (which regulates neutrophil elastase), antithrombin (a central inhibitor of coagulation factors), plasminogen activator inhibitor-1 (PAI-1) (which controls fibrinolysis), [8] and the C-1 inhibitor (which regulates complement activation) [9]. From this perspective, ticks were thought to utilize serpins to interfere with the host’s innate immune defenses [10]. Our group and others have since confirmed that serpins are among proteins that are injected by the tick into the host to regulate feeding and enable pathogen transmission, and for the transmitted pathogens to colonize the host [6,11,12,13,14,15,16].

Serpins are a superfamily of proteins defined by a conserved tertiary structure and a unique mechanism of inhibition: the reactive center loop (RCL) acts as bait for target serine proteases, and upon cleavage, undergoes a conformational change that traps the protease in a covalent complex [7,17]. This is a “suicide inhibition” mechanism that makes serpins irreversible inhibitors, in contrast to many other protease inhibitors. Serpins are astonishingly preserved throughout various species, including animals, plants, and even certain viruses; however, they exhibit a remarkable functional versatility, interacting with a wide range of proteases involved in processes like coagulation, fibrinolysis, complement activation, inflammation, apoptosis, and development [18,19]. Notably, many host protease cascades that serpins can modulate (coagulation/fibrinolysis and the complement/contact (kallikrein–kinin) system) are also among the pathways reported as dysregulated in diseases such as severe COVID-19, where they contribute to endothelial injury and immunothrombosis [20].

A growing number of studies have demonstrated the roles of tick saliva serpins in regulating tick evasion of the innate immune system. For example, *Ixodes ricinus* serpin Iripin-3 strongly inhibits serine proteases kallikrein and matriptase, modulates the adaptive immune response based on reduced survival of mouse splenocytes, impairs proliferation of CD4+ T-lymphocytes, suppresses T-helper type 1 immune responses, and induces regulatory T-cell differentiation [21]. Other tick serpins target thrombin, cathepsin G, and complement components, thereby interfering with coagulation, inflammation, and immune clearance. For example, in *Amblyomma americanum*, salivary serpin 6 inhibits both serine proteases and papain-like cysteine proteases, serpin 19 inhibits the activity of blood clotting factors (including Factor Xa and Factor XIa), while serpin 27 inhibits trypsin and plasmin [22,23,24]. In *I. scapularis*, serpin 17 (*Ixs*S17) inhibits thrombin, trypsin and platelet aggregation: the effectors of the innate immune system. In addition, *Ixs*S17 also inhibits neutrophil elastase and plays a supporting role in dampening early neutrophil/mast cell activity at the bite site. Coupled with this, it interacts with complement system factors protecting the Lyme disease agent (*Borrelia burgdorferi*) from complement-mediated killing, thereby enhancing host colonization [25]. *I. scapularis Ixs*S41 inhibits chymase and cathepsin G (pro-inflammatory proteases released by mast cells and neutrophils that infiltrate the tick feeding site), and protects *B. burgdorferi* from complement-mediated killing [26]. Similarly, s51c10 (1E1 or *Ixs*S6) that is injected into the host at equivalent levels by both uninfected and *B. burgdorferi*-infected ticks [13] is a strong inhibitor of blood clotting factor II (or thrombin), a key effector protease of the blood coagulation system [27]. The relevance of tick serpins to blood feeding is highlighted by experimental studies demonstrating that antibodies against tick serpins shorten attachment time, reduce tick engorgement success or impair pathogen transmission [28,29,30,31,32]. These findings demonstrate that serpins are key players in tick survival and are prime candidates for anti-tick vaccine development.

Despite the importance of serpins in tick biology, knowledge of their genomic organization has remained fragmented. Early work relied on expressed sequence tags (ESTs) or salivary gland transcriptomes, which uncovered dozens of serpin-like-transcripts [33,34,35]. However, transcriptomic surveys alone cannot determine whether serpins occur as isolated genes or in genomic clusters, nor can they capture full exon-intron architectures. Moreover, without chromosomal anchoring, it is difficult to evaluate whether serpins are dispersed across the genome or concentrated in specific loci, subject to duplication and diversification. Such information is crucial because gene family expansions, particularly in clusters, often signal evolutionary “hotspots” of adaptation to ecological pressures [36]. In some arthropods, immune-related and host-interaction gene families (including antimicrobial peptides in *Drosphilla* and salivary gland effectors in mosquitoes) are enriched in tandem clusters, reflecting rapid birth-and-death evolution under host—pathogen arms races [37,38].

We took advantage of the chromosome-level assembly of the *I. scapularis* genome [39] to systematically analyze serpin gene organization. Here we have provided the serpin gene map of 74 serpin genes, of which 73 are encoded on eight of the 15 *I. scapularis* chromosomes with a single serpin gene on unassigned scaffold. Our analysis shows that 70% of *I. scapularis* serpins are encoded on chromosome 10. The significance of the serpin atlas in this study extends descriptive genomics by linking serpin diversity to genomic context, thereby providing a rational framework for prioritizing serpin targets in anti-tick vaccine development. It allows for the prioritization of conserved serpins with crucial roles, which could serve as universal targets. Similarly, it draws attention to rapidly changing or tandemly duplicated serpins, which might be functionally redundant and less appealing for vaccination strategies. Furthermore, the chromosomal context supports comparative genomics among tick species, speeding up the discovery of conserved versus lineage-specific inhibitors [40]. Lastly, the integration of sequence motifs, similarity profiles and expression dynamics across tissues and feeding stages provides insight into functional specialization among *I. scapularis* serpin groups. Outcomes of this manuscript provide the foundation for studies on the functions of serpins in ticks.

## 2. Materials and Methods

### 2.1. Gene Prediction from the Improved I. scapularis Genome

The new chromosome-level *I. scapularis* genome assembly (IscapMGN, GCA_031841145.1) [39] was obtained from the NCBI genome database. For gene prediction, we mapped Illumina paired-end RNA-seq reads from a previous study that sequenced cDNA libraries of blood feeding *B. burgdorferi*-infected and uninfected *I. scapularis* nymphs [41]. Additional reads were sourced from the European Nucleotide Archive (ENA) with query = 6945 and library_strategy = RNA-Seq to download all *I. scapularis* tick RNA-seq reads. Pre-processing of the reads was completed using Trim Galore v0.6.10 (https://doi.org/10.5281/zenodo.7598955, accessed on 13 October 2025) to remove adapters and low-quality reads and SortMeRNA [42] for removal of ribosomal RNA. Mapping of the reads to the genome was achieved using STAR aligner with default parameters [43]. A custom *I. scapularis* “species-excluded” protein database for Arthropoda (taxonomy id: 6656) was curated by downloading all Arthropoda proteins from UniprotKb (https://www.uniprot.org/uniprotkb, accessed on 13 October 2025) and Orthodb (https://www.orthodb.org, accessed on 13 October 2025) databases and excluding *I. scapularis* proteins. We used BRAKER3 pipeline for annotation and gene modelling employing the “species excluded” protein database since the pipeline performs better in gene prediction accuracy with such a protein database [44]. The BRAKER3 pipeline was executed in a local Linux server using the soft-masked genome (as downloaded), mapped RNAseq BAM file, and the custom species excluded protein sequences. The resulting prediction (GTF file) and genome fasta file were viewed in Geneious Prime 2025.2.1 (https://www.geneious.com, accessed on 20 September 2025).

### 2.2. Identification of Serpins from Predicted Genes

Custom Python3-based scripts were created to identify serpins from the predicted genes. Using the Serpin_annotation.py script, the reactive center loop (RCL) sequences from 45 *I. scapularis* serpins that had been identified and characterized in a previous study in our lab [33] were used to identify protein sequences containing RCL sequences within their C-terminal regions. The script systematically parses the last 100 amino acids from the C-terminus of a predicted protein (“C-terminal window”), comparing each RCL sequence to every possible protein subsequence of similar length within this window, permitting up to 30% mismatches (with additional control to prevent more than one consecutive mismatch). For each protein, only one best motif match (fewest mismatches, or, in case of ties, the earliest occurrence) is retained. Finally, all protein sequences harboring qualifying RCLs are output as identified serpins in a FASTA file, and their RCL match coordinates are written in a GFF3 file. The Serpin_gtf_filter.py script is then used to extract the RCL-containing proteins from the BRAKER3 GFF file while annotating the RCL sequence, resulting in a GTF coordinates file locating each possible serpin (this makes it easier to view the predicted serpins in Geneious prime or any other viewer). The RCL sequences from all predicted serpins and the initial 45 RCL sequences were concatenated and used for another round of serpin prediction, to make sure all possible serpins were identified. Finally, each identified serpin gene was manually checked to confirm the start /stop codons, exons, introns and the ‘NAVYFKG’ N-terminal hinge sequence present in serpins [45]. For manual gene modeling, each serpin sequence was annotated using Blastp (https://blast.ncbi.nlm.nih.gov/Blast.cgi, accessed on 13 October 2025) and a conserved domain database search (https://www.ncbi.nlm.nih.gov/cdd, accessed on 13 October 2025) to confirm serpin identity and presence of the RCL. Additionally, for hits that did not match the blast subject fully (partial predicted genes), a check of the gene model was conducted to locate the missing exon match by looking downstream or upstream of the gene location either manually or using Python scripts (script_find_serpin_sequence_given_DNA.py or map_protein_peptide_to_genome.py). To synchronize the identities of serpins identified in this study and the 45 serpins from the previous study, all identified serpins were renamed with their closest matches using the ‘Find_and_rename_serpins_with_old_names.py’ script. The final syntax adopted to name serpins is by serpin number and the chromosome number (numbering starts from chromosome 1). Signal peptide prediction was completed using SignalP 6.0 [46] and prediction of molecular weights and isoelectric point (pI) was performed using custom Biopython scripts.

### 2.3. Gene Structure and Orientation Analysis, Motif Search, and Pairwise Alignment

Gene structures (exon–intron organization) were characterized using the official *I. scapularis* genome annotation and the curated serpin gene list used throughout this study. For each serpin locus, exon coordinates were extracted and ordered by genomic position; exon number, exon lengths, intron lengths, and total gene span were computed from the outermost transcript boundaries. When multiple transcript isoforms were annotated for a locus, we used the longest coding transcript as the representative model to avoid double-counting gene architecture, and we retained isoform-specific differences for supplementary reporting. Gene orientation was assigned directly from the annotated strand field (+/–) for each serpin locus, and cross-validated by confirming that transcript coordinates progressed in the expected 5′ to 3′ direction for the assigned strand. For tandem-array orientation patterns, adjacent serpin loci within the same cluster were classified as head-to-tail (same strand), head-to-head (convergent; opposite strands with 5′ ends facing), or tail-to-tail (divergent; opposite strands with 3′ ends facing) based on the relative positions of gene start and end coordinates. Strand bias across the full serpin repertoire was tested with an exact binomial test under a null of equal probability for + versus − strands, and orientation-category enrichment within clusters was summarized.

For motif discovery, we implemented a multi-stage, motif-based clustering pipeline (https://github.com/AlexGaithuma/Identification-of-Serpins-from-whole-genome/tree/main/Find_motif_pipeline, accessed on 20 February 2026) to identify conserved and adaptive sequence features across serpin proteins. Full-length serpin amino acid sequences, multiple-sequence alignments, curated N-terminal segments, and RCL regions were first preprocessed to standardize sequence identifiers across all inputs, ensuring one-to-one tracking of each sequence across contexts. Global de novo motif discovery was then performed on the full-length sequences using MEME [47] to generate an unbiased catalog of serpin motifs. Using a custom Python workflow (extract_contexts.py), we derived biologically meaningful context windows for each sequence, including N-terminal segments, RCL loops, gap-stripped full-length sequences, and high-entropy “variable” windows identified from MAFFT-aligned sequences by sliding-window Shannon entropy. In parallel, local windows centered on each motif instance reported in the global MEME output were extracted to capture both motif cores and flanking residues. For every context FASTA, MEME was rerun to obtain context-specific motif models, which were then scanned back against the full sequence set with FIMO (MEME-suite) to construct a binary sequence-by-motif occurrence matrix (build_motif_matrix.py). This matrix was standardized and subjected to a two-step clustering procedure (cluster_by_motifs.py), in which initial agglomerative clustering provided provisional groups that were used to train a random forest classifier; motif feature importances from this classifier were then used to weight motif columns before a final round of agglomerative clustering, enriched for biologically discriminative patterns. Motif-level statistics (width, e-value, information content, sequence and group entropy), consensus sequences, and per-motif instance FASTA files were collated with summarize_motifs.py, which also generated sequence logos and a motif–motif overlap network. Finally, the weighted motif matrix and cluster assignments were visualized in R Statistical Software (v4.4.0; R Core Team 2024) as heatmaps and group annotation plots. 

For pairwise alignment analysis, all serpin sequences were aligned in an all-vs-all pairwise comparison using both global and local alignment. For every pair, a Needleman-Wunsch global alignment was performed, reporting the global percent identity as the fraction of identical residue pairs across the full alignment length, including positions aligned to terminal gaps. In parallel, a BLASTP-style Smith–Waterman local alignment was computed to capture the highest-scoring local similarity region between the same pair of sequences. From this local alignment, the local percent identity, the length of the aligned ungapped region, and the start and end coordinates of the optimal local alignment in the query sequence were extracted. All metrics (sequence IDs, global identity and alignment length, local identity, local alignment length, and query start/end positions) were written to a tab-delimited file.

### 2.4. In Silico Prediction of Function Based on Reactive Center Loop Amino Acids

To further characterize the serpins, we predicted their role and function based on the RCL residues. RCL residues were identified in the multisequence alignment based on published data [7,48]. The substrate positions were numbered using the Schechter-Berger nomenclature and included the hinge residues (P15-P9) and specificity core (P4-P4′). The predicted primary target proteases were assigned by P1 residue (the scissile-bond residue that inserts into the target protease’s S1 pocket). In serine protease biochemistry, P1 Arg/Lys is a hallmark of trypsin-like targets (coagulation factors, kallikrein, trypsin-like complement proteases), hydrophobic/aromatic P1 residues (Phe/Tyr/Trp/Leu/IIe/Val/Met) point to chymotrypsin-like enzymes (e.g., cathepsin G, mast-cell chymase), and small neutral P1 residues (Ala/Ser/Gly/Val) point to elastase-like enzymes. Where P1 = Val/IIe with small P1′ (Ala/Ser/Gly/Thr) these serpins were flagged as inhibiting “Dual”, suggesting chymotrypsin-like/elastase-like protease preference despite a hydrophobic P1. Additionally, from the P1 Arg/Lys classification, we projected pathway context: the classical and lectin complement initiators (C1r, C1s, MASP-1/MASP-2) are themselves trypsin-like, so a serpin with a trypsin-like P1 is structurally predisposed to inhibit coagulation proteases and/or early complement proteases that cleave after basic residues. These assignments follow established S1 pocket preferences and large-scale profiling datasets [49]. Because serpin inhibition hinges on a metastable fold, RCL insertion, and exosite contacts, we treated P1-only cells as screening-level predictions, and we flagged non-canonical P1 residues (e.g., Asn, Glu) as unknown specificities. There is evidence that residues P4–P2 and P1′–P3′, loop flexibility, and cofactors can shift or broaden specificity beyond the signal carried by P1 [8]. For pathway assignment, serpins with P1 Arg/Lys, were marked as coagulation/complement controllers; serpins with P1 hydrophobic/aromatic were annotated as neutrophil/mast-cell inflammation controllers; and serpins with small P1 residues annotated as tissue protection and inflammation controllers. Where experimental studies have been performed in our lab (S12c5, S13c5, S31c10, S45c10, S48c10, S49c10, S51c10 and S61c10), we updated the predicted roles to reflect concordance or mixed specificity. This recognizes that some serpins naturally co-inhibit across protease classes, as reported for multi-target serpins [50]. Overall, our assignment pipeline integrates: (i) P1-anchored serine protease rules; (ii) complement-specific evidence for trypsin-like early proteases; and (iii) RCL/exosite caveats from serpin mechanisms [51].

### 2.5. Expression Analysis of I. scapularis Serpins

To determine the expression of *I. scapularis* serpins during feeding on mammalian hosts, we used RNAseq raw reads from an earlier study in our lab (same RNAseq reads used for annotation) [41]. Briefly, 20 nymphs were placed on each ear patch of two rabbits. In one rabbit, *B burgdorferi*-infected *I. scapularis* nymphs were placed while in the other, uninfected nymphs were placed. The ticks were monitored until more than 12 ticks per ear patch were attached to the skin and unattached ticks removed. Sampling of feeding ticks was completed at 24, 48, and 72 h of attachment to the host skin, when three individual ticks (biological replicates) were sampled from each ear at each time point. Each tick produced three samples (midgut, salivary gland and carcass). Three ticks per ear patch were allowed to feed until engorgement, and then collected and dissected after they self-detached from the skin. For every six ticks sampled per time point, organs from two ticks (one from each ear) were pooled to make three replicate samples per time point. Total RNA was isolated from replicate tick organs and prepared for sequencing on an Illumina NovaSeq 6000 (Illumina, San Diego, CA, USA) as per manufacturer’s protocol for total RNA sequencing. Enzymatic rRNA depletion was first completed to remove rRNA, then fragmentation into smaller, uniform pieces was performed. Random hexamers were then used in synthesis of the first strand, followed by second strand synthesis, to create a cDNA library. The cleaned-up library was sequenced in 150 bp paired-end runs. The raw reads were mapped to chromosomes containing the serpins using STAR aligner with an additional GTF file containing the coordinates of each serpin. Serpin gene counts were imported from a featureCounts table [52] and paired with sample metadata, ensuring consistent sample naming and filtering across analyses. We used DESeq2 in R to model counts with a negative binomial framework and to estimate library size factors and dispersions, fitting time-course contrasts within each organ and infection status (design~time) and extracting log2 fold-changes with multiple testing-adjusted *p* values. First, to quantify deployment over feeding time and organs, we used a single DESeq2 model that adjusts for infection but tests tissue and time as the main signal: counts were modeled as a function of treatment + organ + time + organ:time. We used a likelihood-ratio test (LRT) to test whether organ:time improves model fit, because that test directly answers the question of whether the time course differs by organ without fragmenting the analysis into many pairwise comparisons. We reported false discovery rate (FDR) adjusted *p* values using the Benjamini–Hochberg method. Second, to quantify infection-associated effects in a way that respects the study design, we estimated infection log2 fold-change (infected vs. uninfected) within each organ at each timepoint using a model that allows organ × time × treatment structure. For inference, we summarized how many organ–time cells yielded evaluable effect sizes and how many passed FDR thresholds. We performed organ-specific time contrasts while still adjusting for infection. Within each organ, we fit treatment + time and tested 48 h vs. 24 h, 72 h vs. 24 h, and SD vs. 24 h. We also defined a biologically clear stage grouping, early (24–48 h) vs. late (72–SD), and tested late vs. early. Genes were called early-deployed if late–early log2FC < 0 (at Minimum Detectable Effect at 80% power: MDE80) with FDR < 0.05, and late-deployed if late–early log2FC > 0 with FDR < 0.05. Finally, to identify serpins deployed similarly across organs, we computed normalized expression from the count matrix and metadata, summarized mean expression per gene per organ on the log2 (normalized counts + 1) scale, and combined this with organ-wise trajectory similarity across time.

All results were summarized in an Excel workbook and multi-panel PDFs generated for each organ across time-point comparisons. To visualize global expression structure, normalized/variance-stabilized serpin expression was z-score scaled and displayed as annotated heatmaps using pheatmap, with columns ordered by experimental factors rather than clustered. Sample-level structure was further assessed by PCA (prcomp) on variance-stabilized serpin expression and visualized as 2D hull plots. All analyses were completed using custom scripts in R program.

## 3. Results

### 3.1. Most I. scapularis Serpins Are Glycosylated Secreted Proteins Encoded by Intronless Genes

**Figure 1:**
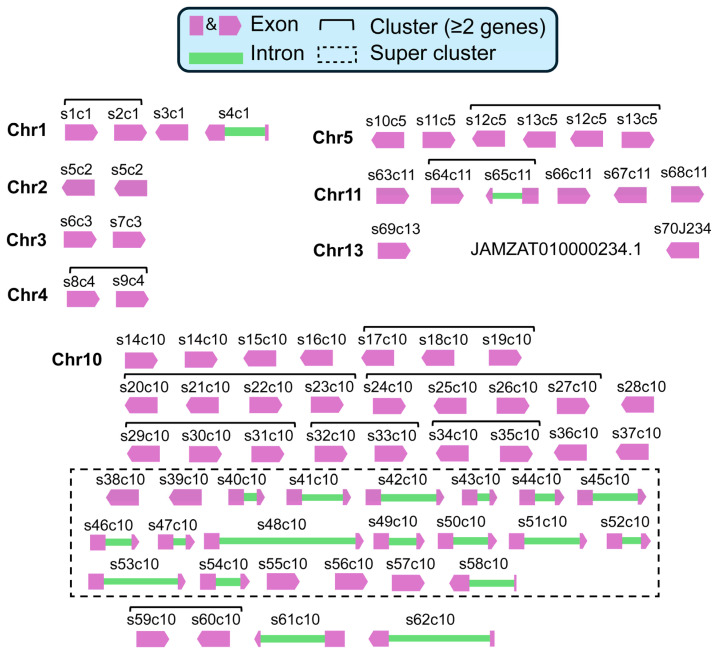
Chromosomal distribution and naming scheme of *I. scapularis* serine protease inhibitors. Serpin loci (labels in the format S#C#, where S = serpin ordinal along the genome and C = chromosome) are mapped across Chr1, Chr2, Chr3, Chr4, Chr5, Chr10, Chr11, Chr13 and one unplaced scaffold (JAMZAT010000234.1). Each locus is drawn as an arrow; arrow direction indicates transcriptional strand (► forward/plus, ◄ reverse/minus). Introns are represented by green rectangles with length to scale. Brackets/blocks denote tandem clusters (defined in Methods), which are frequently strand-biased; a prominent hotspot is evident on Chr10, where long, colinear arrays predominate. Intronic distances are to scale in panels; inter-cluster gaps are not to scale but truncated for clarity.

From the *I. scapularis* genome, a total of 74 serpin genes (including copies) were identified in eight of the 15 chromosomes. We named the serpins based on the chromosome in which they are found (e.g., S1c1), where “S” denotes the serpin identifier (numbered sequentially along the genome) and “c” identifies the chromosome (Figure 1, Supplementary Tables S1 and S2). Global pairwise protein sequence comparisons across the serpin set identified four serpins present as exact duplicate copies (S5c2, S12c5, S13c5, and S14c10), showing 100% global amino-acid identity to their corresponding partner sequence (Supplementary Table S3). In addition, nine recent paralog pairs met a stringent similarity threshold of ≥98% global identity: S55c10/S56c10, S5c2/S15c10, S21c10/S23c10, S22c10/S23c10, S59c10/S60c10, S18c10/S26c10, S21c10/S22c10, S15c10/S70j234, and S5c2/S70j234. The length of the serpins ranged from 372 to 455 amino acids (mean = 396 aa, SD = 11), consistent with the published amino acid core region size of serpins [53]. 

The calculated molecular weights of serpins ranged from 41 to 51 kDa and isoelectric points ranging from 5.1 to 9.8 (median = 6.6). All except one serpin (S64c11) had the N-glycosylation motif (Asn-X-Ser/Thr, where X ≠ Pro) and we found signal peptide sequences in 63 serpins (85%) (Supplementary Table S4), indicating that most serpins are secreted, highlighting their role in tick saliva. We identified 20 serpins (28.5%) as multi-exon genes, each with a two-exon organization with a highly conserved splice junction at the protein level (consensus ≈ TVFLPK|FKLETKYSL), indicating a shared ancestral intron position rather than independent intron gains. In all but two serpins (S65c1 and S61c10), exon 1 encodes the conserved N-terminal motif (NAIYFKG), whereas exon 2 encodes the downstream region that includes the RCL, consistent with a modular architecture that separates a conserved N-terminal scaffold from the specificity-determining inhibitory loop. The two outliers, S65c1 and S61c10, require experimental confirmation of the exon architecture. The rest of the genes were intronless genes (Figure 1 and Supplementary Table S1). Exon lengths ranged from 137 to 1382 bp (mean = 931 bp, SD = 358) and intron lengths from 498 to 7613 bp (mean = 2201 bp, median = 1494 bp, SD = 1742). 

### 3.2. I. scapularis Serpin Genes Occur as a Tandem Array of Gene Clusters

We found highly uneven distribution of serpin genes in the genome, with chromosome 10 harboring most serpins (50 serpin genes, 67.6%), followed by chromosomes 5 and 11 (each six serpins), and chromosome 1 (four serpins) (Figure 1). Chromosomes 2, 3, and 4 each carried two serpins, while chromosome 13 and the unplaced contig JAMZAT010000234.1 each had a single serpin. In total, there were 69 unique serpins in eight chromosomes and one serpin in an unplaced contig. The mean number of serpins per chromosome was 4.7; however, because chromosomes differ substantially in size and gene content, we did not test against an equal per-chromosome expectation. As a sensitivity analysis, we evaluated chromosomal non-randomness using a χ^2^ goodness-of-fit test in which the expected number of serpins on chromosome *i* was proportional to chromosome length ($E_{i}=N_{\mathrm{serpin}}\times L_{i}/\sum L$; $N_{\mathrm{serpin}}=69$ serpins on chromosomes); one additional serpin gene occurred on an unplaced scaffold and was excluded from this length-weighted test. The observed serpin distribution remained strongly non-random (χ^2^ = 655.549, df = 14, *p* = 7.83 × 10^−131^; Supplementary Table S5), supporting the idea that true chromosomal concentration extends beyond differences in chromosome size alone.

**Figure 2:**
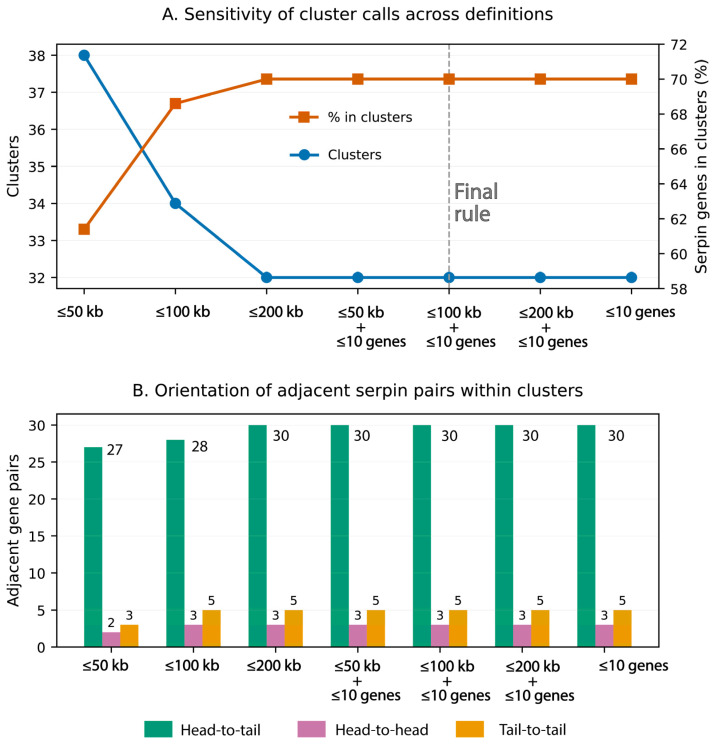
Serpin clustering robustness and orientation patterns. (**A**) Sensitivity of inferred serpin gene clusters to alternative operational thresholds. The blue line shows the total number of clusters detected under each threshold, and the orange line shows the percentage of serpin genes assigned to clusters. Thresholds tested include distance-only rules (≤50 kb, ≤100 kb, ≤200 kb) and distance plus ≤10 intervening genes. The dashed marker indicates the final definition used in the manuscript (≤100 kb plus ≤10 intervening genes). (**B**) Orientation of adjacent serpin gene pairs within clusters in different distances plus ≤10 intervening genes. Bars show counts of head-to-tail (tandem), head-to-head (convergent), and tail-to-tail (divergent) arrangements for immediately neighboring serpin loci within clusters; numbers above bars indicate observed counts. Full cluster membership, summary statistics, and the threshold sensitivity results are provided in Supplementary Table S5.

Strand orientation was assigned at the gene level from the genome annotation, showing that 46 serpin genes (62%) are encoded on the positive strand and 28 (38%) on the negative strand, indicating a significant strand bias (exact binomial test, $P=4.7\times 10^{-2}$). Cluster robustness was checked by repeating the analysis with several simple cutoffs (≤50 kb, ≤100 kb, ≤200 kb, and ≤10 intervening genes). When we loosened the distance cutoff, the number of clusters dropped from 38 (≤50 kb) to 32 (≤200 kb), while the share of serpins assigned to clusters rose from 61.4% to 70.0% (Figure 2A; Supplementary Table S5). Importantly, once we required ≤10 intervening genes, our results stabilized: the same 32 clusters and 70% clustered serpins were recovered across the tested distance windows (50–200 kb), and even when the distance limit was removed. We therefore define serpin clusters in *I. scapularis* as a set of serpin genes on the same chromosome where each neighboring pair is within ≤100 kb (boundary-to-boundary) and separated by ≤10 intervening annotated genes (including non-serpin genes). We chose 100 kb because it is the simplest distance cutoff that matches the plateau seen in our sensitivity analysis, and because arthropod studies commonly define tandem gene arrays using the same “≤10 intervening genes” idea [54]. We avoid using a larger distance cutoff as the primary definition because duplicated blocks in many genomes can span up to ~200 kb, so a wider window is, in principle, less specific for tight local arrays. Notably, 49 serpin genes (70% of all serpins) are found within clusters, pointing to the prevalence of local gene duplication. Orientation analysis showed that most clustered serpin genes occur in tandem (head-to-tail) configuration (30 pairs), while those arranged head-to-head (three) or tail-to-tail (five) were fewer (Figure 1 and Figure 2B). This structural orientation and dense local clustering are consistent with tandem duplication as the primary mechanism of serpin gene expansion, and occasional inversion events to introduce structural diversity.

### 3.3. Chromosome 10 Is the I. scapularis Serpin Innovation Hotspot

Chromosome 10 stands out as the dominant center of serpin innovation within the *I. scapularis* genome, containing 50 serpin genes, making it the dominant center of serpin expansion. These genes form 17 discrete clusters, ranging from 2 to 21 genes per cluster (Supplementary Table S5). Focusing on chromosome 10, the serpin complement comprises 49 serpins arranged into 17 physical clusters, including nine singletons, three 2-gene clusters, three 3-gene clusters, one 4-gene cluster, and a single 21-gene supercluster (Cluster CM063355.1_24; 37) which spans 50,307,207–50,760,803 bp (~0.45 Mb) and contains 21/49 (42.9%) of all chromosome 10 serpins (S38c10-S58c10) arranged mostly in tandem orientation (mean intergenic distance = 11,908 bp; range = 679–43,544 bp). The average within-cluster intergenic spacing across the chromosome is 38,912 ± 27,501 bp, several-fold shorter than the genome-wide mean for tick genes (~ 220 kb in *I. scapularis*), confirming that these genes are densely and non-randomly packed. Orientation analysis show that ≈ 83% of adjacent pairs are aligned head-to-tail, 11% tail-to-tail, and 6% head-to-head, a pattern consistent with tandem duplication. Some rearrangements, including the mixed tandem/head-to-head structure in cluster S24c10–S25c10–S27c10, point to irregular and uneven crossing-over and inversion, which have both been shown to speed up gene-family turnover [55,56].

Sequence identity comparisons across the serpin repertoire identified four serpin loci present as exact duplicate pairs (S5c2, S12c5, S13c5, and S14c10), with no amino acid differences across the full-length global alignment between each duplicate copy (Supplementary Table S3). In addition, nine high-identity paralog pairs were detected with ≥98% global protein identity, comprising S55c10/S56c10, S59c10/S60c10, S21c10/S22c10, S21c10/S23c10, S22c10/S23c10, S18c10/S26c10, S5c2/S15c10, S15c10/S70j234, and S5c2/S70j234. Notably, 8 out of 9 (88.9%) of these ≥98% identity relationships included at least one chromosome 10 serpin, while the remaining relationship linked non-chromosome 10 serpins. All-by-all global protein comparisons among the 49 chromosome 10 serpins represent 1176 pairwise alignments (49 × 48/2); within these, 60/1176 (5.1%) comparisons fell within 70–97% global identity and were classified as diverged paralog pairwise relationships, whereas the remaining 1116/1176 (94.9%) within-chromosome comparisons were <70% identity and were therefore treated as non-homologous at the level of global protein alignment.

Together, these observations indicate that serpin sequence similarity spans a broad range, from exact duplicates (100% identity) through near-identical paralogs (≥98%) to moderately diverged paralogous relationships (70–97%), while the majority of other chromosome 10 serpin–serpin comparisons remain below 70% identity. The simultaneous presence of exact duplicates and multiple tiers of divergence is consistent with serpin gene duplication and subsequent sequence diversification occurring at different apparent stages within the genome. In chromosome 10, the dense physical organization of several serpin clusters, most notably the large 21-gene region with short intergenic spacing, supports the interpretation that local duplication has contributed to serpin accumulation in this chromosome.

### 3.4. Predicted Functional Profiles of I. scapularis Serpins Point to Broad and Partially Redundant Roles

To predict the functions and substrates of the serpins, the active domain of serpins (the RCL) is used as a screening predictor. The predicted functions of serpins fall into three major functions: (1) Blood clotting and complement inhibition—inhibition of thrombin, kallikrein, factor Xa, Factor XIa, trypsin-like complement proteases; (2) inflammation control—inhibition of neutrophil/mast cell proteases like cathepsin G, chymase, and chymotrypsin; and (3) tissue protection—inhibition of neutrophil elastase and proteinase 3. Each of the identified functions was predicted using RCL residues as a criterion (Table 1) for use as a screening tool to categorize the serpins. The serpins with P1 amino acid residues containing Arg/Lys were classified as blood clotting and complement inhibitors, maintaining blood-flow and reducing opsonization at the tick feeding site. Structural and enzymology studies are consistent in classifying complement proteases involved with blood clotting as trypsin-like, supporting our prediction criteria [50]. Serpins with P1 residues containing aromatic/hydrophobic amino acids at their P1 sites were classified as inhibition of neutrophil/mast cell proteases functioning to dampen inflammation, limit protease-activated receptor (PAR) signaling, restrain cytokine release, and reduce matrix proteolysis [48]. Serpins with small amino acid residues such as Gly/Ala/Ser/Val at the P1 site were classified as inhibitors of neutrophil elastase with a tissue protection role, preventing damage from neutrophil degranulation and modulating secondary inflammation. This was inferred from evidence showing neutrophil elastase proteinase 3 inhibitors favoring small, neutral P1 residues [57].

Our in silico predictions align closely with empirically validated serpin functions. Notably, serpins whose targets have already been experimentally confirmed support the robustness of our criteria for functional prediction. Serpin S51c10 (formerly IxsS6 or 1E1) was confirmed to inhibit thrombin (as well as trypsin, cathepsin G, and Factor Xa) [27], agreeing with our prediction of it as a strong blood coagulation inhibitor. S61c10 (formerly IxsS17) was confirmed to inhibit trypsin-like proteases (trypsin, trypsin IV, and factor Xa) and inhibited complement system serine proteases C1s, C2, and factor I [25], agreeing with our prediction of it as a strong blood coagulation inhibitor. S13c5 (formerly IxsS19) has been shown to inhibit trypsin IV, alpha trypsin, blood clotting factor (f) Xa, fXIa, and fIXa, plasmin, chymase and cathepsin G, making it a broad anti-hemostatic/anti-inflammatory and complement inhibitor, while our prediction placed it as a strong blood coagulation inhibitor [57]. Similarly, serpin S45c10 inhibits porcine elastase, chymotrypsin, human chymase, and cathepsin G in vivo and is therefore an inflammation modulator with tissue protection functions. This agrees with our prediction in this study. In contrast, S48c10, which has an identical RCL to S45c10, inhibits kallikrein, chymotrypsin, human chymase, and cathepsin G, making it an inflammation modulator with anti-coagulation functions [59]. These two serpins show that other residues outside the RCL contribute to target diversity while maintaining functional specificity, thus broadening the function of serpins in *I. scapularis*. The four serpins (S61c10, S13c5, S45c10 and S48c10) may be evidence that these serpins can both straddle multiple functions and be broadly functional (inhibiting both blood clotting and toning down inflammation), exceeding and expanding our prediction borderlines. This is consistent with serpin functional mechanisms and RCL-context effects [8]. It is expected, since in RCL dynamics, P2–P1 contexts frequently expand or shift specificity, and some serpins are naturally polyvalent [61].

Some of our predictions did not agree with experimental evidence (when RCL P1 residues are small): our prediction of S12c5 (formerly *Ixs*S20) as an inhibitor of neutrophil elastase and proteinase 3 disagrees with experimental data that shows it inhibits chymotrypsin, human chymase and cathepsin G, placing it as an inflammation control rather than a tissue protection serpin [58]. This points to a greater diversification of functional breadth beyond the criteria that protease inhibitors with small P1 residues are inhibitors of neutrophil elastase and proteinase 3. The P1-based prediction can point researchers in the right direction in determining the function of *I. scapularis* serpins, but experimental studies remain the gold standard to elucidate physiological functions.

### 3.5. Feeding Stage and Tissue-Specific Deployment of I. scapularis Serpins During Blood Meal Acquisition

**Figure 3:**
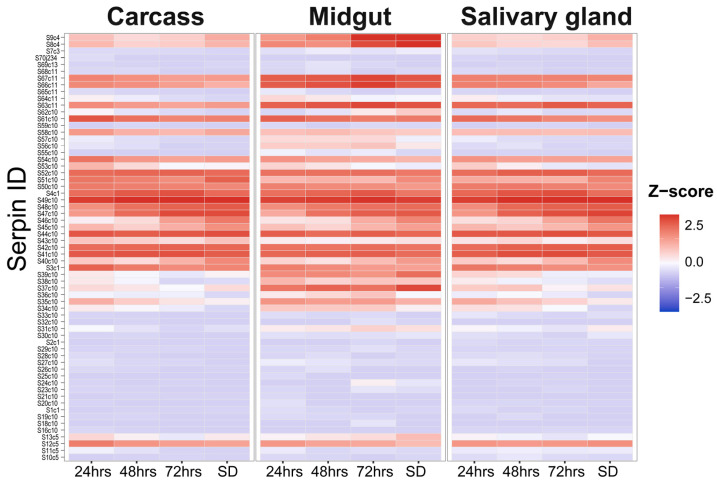
Serpin deployment across organs and feeding time (infection-agnostic). Heatmap shows serpin expression in carcass, midgut and salivary glands at 24 h, 48 h, 72 h and the terminal feeding timepoint (SD). For each serpin, cells represent the mean log2(DESeq2 size-factor normalized counts + 1) for each organ–time condition, averaged across infection status (infected and uninfected samples pooled based on the statistical outcome that infection had no large effect on expression). Colors depict a global Z-score computed across all serpin-by-condition means, enabling comparison of color intensity between genes and across organs/time (red = above the global mean; blue = below the global mean).

To answer the question of how serpins are expressed, we focused on how *I. scapularis* ticks deploy serpins across feeding and tissues and asked whether *B. burgdorferi* infection affects expression. Gene expression profiling across feeding time stages (24, 48, 72 h and replete fed self-detached [SD]), and tissues (salivary glands, midgut, and carcass) reveal that *I. scapularis* serpins are not uniformly expressed, but instead follow clear patterns driven mainly by feeding progression (Figure 3). Out of the 70 serpins, 65 serpins had < 1 reads mapped to them, indicating expression during tick feeding. Only five serpins (S5c2, S6c3, S14c10, S15c10, S60c10) were not expressed. Following standard differential analysis procedure, 58 serpins had a total count of five reads for all samples and were used for differential analysis. Of these, 35 serpins were differentially expressed in any comparison (between any two tissue samples and between any two timepoints of feeding; design formula = organ + time + treatment + organ:time + organ:treatment + time:treatment + organ:time:treatment). This analysis revealed the following differentially regulated genes: S3c1, S4c1, S8c4, S9c4, S12c5, S13c5, S31c10, S34c10, S35c10, S36c10, S37c10, S38c10, S39c10, S40c10, S41c10, S42c10, S43c10, S44c10, S45c10, S46c10, S47c10, S48c10, S49c10, S50c10, S51c10, S52c10, S53c10, S54c10, S56c10, S58c10, S61c10, S62c10, S63c11, S66c11, S67c11. The remaining 22 genes (S1c1, S2c1, S7c3, S10c5, S11c5, S16c10, S19c10, S23c10, S24c10, S26c10, S27c10, S28c10, S29c10, S30c10, S33c10, S55c10, S57c10, S59c10, S64c11, S65c11, S68c11, S69c13) did not show any significant differential expression.

**Figure 4:**
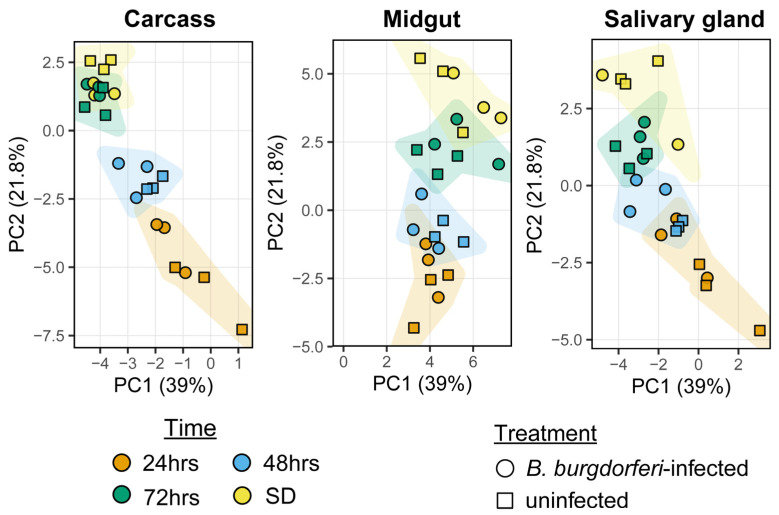
Principal component analysis of serpin expression across tissues and feeding stages. Principal component analysis (PCA) was performed using normalized RNA-seq expression values of the 35 differentially expressed *I. scapularis* serpin genes across feeding stages and tissues. Samples are shown as individual points and are faceted by tissue: carcass, midgut (MG), and salivary glands (SG). Point and convex hull color indicates feeding stage (24 h, 48 h, 72 h, and self-detached [SD]), and point shape denotes treatment (*B. burgdorferi*-infected or uninfected). PC1 and PC2 represent the first two principal components and capture the largest fractions of variance in serpin expression within each tissue.

Quantitative analysis on organ-by-time vs. the effect of *B. burgdorferi* infection shows that serpin expression is organized as a time- and tissue-dependent deployment program during feeding, while infection contributes little consistent signal in this dataset. It is important to note that sensitivity is not uniform across the gene family: the median abundance was low (median baseMean = 7.66), and 46.03% of serpins had baseMean < 5, which reduces the power to detect subtle infection differences for nearly half the family. This data supports no broad infection-driven serpin program, but they do not rule out small or cell-type specific effects. The organ-by-time deployment test was strong: 20 serpins were significant by the organ-by-time likelihood ratio test (LRT) at false discovery rate (FDR) < 0.05 (21 serpins at FDR < 0.10). By contrast, infection-associated effects had a much smaller effect: only 10 of 756 evaluable infected versus uninfected organ–time comparisons (1.32%) reached FDR < 0.05 (Supplementary Table S6 and Figure S1). This pattern fits a model in which feeding physiology dominates serpin regulation, while infection-driven effects, if present, are small, heterogeneous, or limited to specific contexts. Principal component analysis (PCA) of the 35 differentially expressed serpins also supports the inference that the tick feeding timepoint and tick organs are the primary axes structuring serpin expression, with minimal contribution from infection status (Figure 4). This temporal trajectory is most pronounced in the midgut where late feeding samples (72 h and SD) shift strongly, consistent with coordinated transcriptional changes during engorgement. When examined separately by tissue (salivary gland, midgut and carcass), samples show clear temporal separation between 24, 48, 72 h and self-detached (SD) time points. This indicates a programmed progression of serpin expression during blood feeding rather than random fluctuation. These global patterns indicate organ context and feeding progression dominate serpin expression dynamics. In contrast, infected and uninfected samples overlap extensively at each timepoint in all tissues, demonstrating that infection status explains little of the variance captured by the major components.

As a purely predictive measure for generating hypotheses for further studies, we make some hypothetical observations for differentially expressed serpins. The most reproducible signal is the feeding stage-dependent increase in a subset of serpins that have basic amino acid residues at their P1 in the midgut during late feeding (basic P1 = arginine/lysine: characteristic of inhibitors targeting trypsin-like serine proteases including thrombin, factor Xa and kallikrein-like enzymes). Within this class, S8c4, S9c4, S13c5, S31c10, S37c10, S39c10, S46c10, S47c10, S49c10, and S66c11 seem to show low-to-moderate expression at 24–48 h, followed by a consistent increase at 72 h and/or SD in the midgut. In contrast, other basic P1 serpins S51c10, S52c10, S53c10, S54c10, S58c10, and S61c10 (IxsS17) do not show a visible late feeding increase and instead remain lower or stable across feeding stages (Supplementary Figure S1). This pattern may indicate that late midgut enrichment is not a universal property of all basic P1 serpins, but rather a feature of a defined subset pointing to functional diversification within this class. Nevertheless, the recurrence of late feeding midgut-increased expression is consistent with the increased need to regulate host-derived proteases within the expanding blood meal in the midgut which intensifies sharply as the ticks approach engorgement. Although ticks show numerically higher expression for individual genes, error bars overlap extensively between infected and uninfected groups, and statistical power is low; therefore, no infection-specific conclusions are supported by the data and further experimental studies are needed to investigate this variable.

In contrast, we hypothetically deduce that serpins with small P1 residues (Ser/Gly/Ala), linked to elastase and inflammation-associated proteases, seem to show heterogenous and gene-specific trajectories rather than a unified pattern. This group, S4c1, S12c5, S34c10, S35c10, S36c10, S38c10, S42c10, S56c10, S62c10, and S63c11, exhibits modest expression changes across feeding time with slight midgut increases near 72 h or constant across tissues. This group may point to a lack of synchronized induction, and thus be suggestive of localized or context-dependent inflammatory control. Lastly, serpins with hydrophobic or aromatic P1 residues associated with chymotrypsin-like proteases, S1c1, S2c1, S3c1, S5c2, S6c2, S7c2, S10c4, S11c4, S14c5, S15c5, S16c5, S18c6, S19c6, S20c6, S21c6, S22c7, S23c7, S24c7, S25c7, S26c7, S27c8, S28c8, S29c8, S30c8, and S32c9, seem to display broader tissue distributions and moderate late feeding increases across tissues rather than a strict midgut restriction. Together, these results point to a suggestion that time and tissue are the dominant axes shaping serpin expression, while the P1 residue class predicts tendencies rather than uniform behavior.

### 3.6. I. scapularis Serpins Are Highly Diverse but Structurally Conserved as Revealed by Consensus Amino Acid Motif Profiling

**Figure 5:**
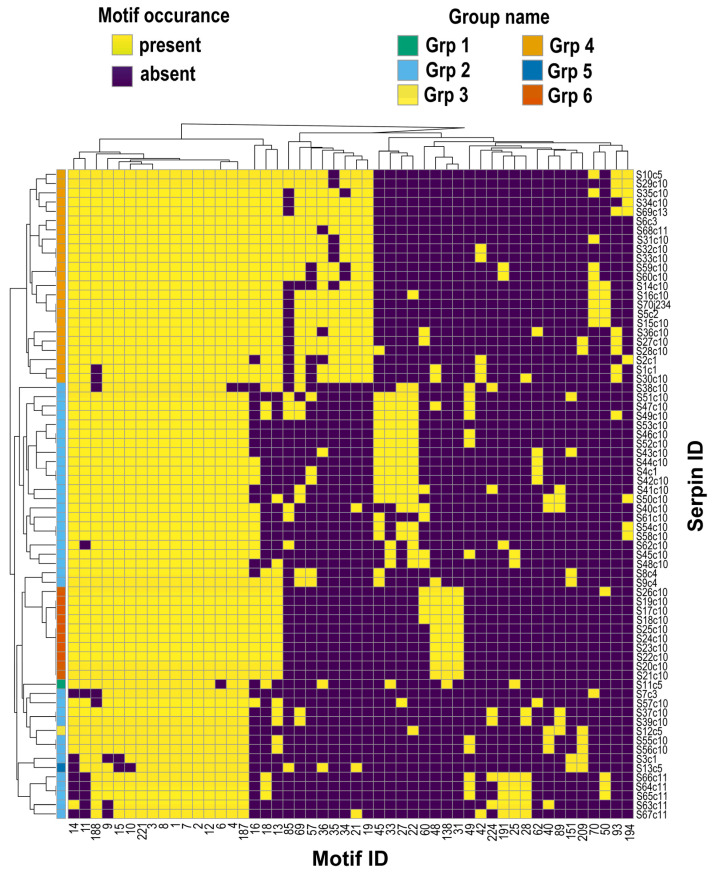
Motif profiles and serpin grouping based on consensus amino acid motifs. The heatmap shows 73 consensus motif sequences occurring in more than 10 serpin sequences mapped according to absence (0; yellow) and presence (1; purple). The serpins are then clustered into groups based on the motifs they harbor.

We identified 50 motif sequence patterns, and, based on the occurrence of these motifs, clustered the serpins into six groups (Figure 5 and Supplemental Table S7). Each consensus amino acid sequence motif occurred in more than 10 serpins across the *I. scapularis* genome, including the prominent N-terminal–RCL motif (NAIYFKG: Motif_187) that is present in 70 serpins, and a companion RCL-core signature (EEGTVAAAATGVVIVR: Motif_188) that is present in 63 serpins. This indicates that the serpin repertoire is built from a relatively small but highly reused structural sequence “vocabulary”. This emphasizes that the *I. scapularis* serpins represent an expanded and diversified arsenal built on a conserved structural scaffold. This specifically points to a conserved inhibitory “center” within the RCL that likely underpins shared target preferences. Since the RCL is the principal determinant of serpin-protease specificity and is solvent-exposed in the native form, enrichment of RCL-adjacent motifs in our data suggest optimization of RCL chemistry and dynamics for target coverage [62]. We identified 16 motifs (Figure 4; first 16 motifs from left) that occur in 58–70 serpins, namely motifs 1, 2, 3, 4, 6, 7, 8, 9, 10, 11, 12, 14, 15, 187, 188 and 221, showing overlapping motif signatures.

## 4. Discussion

Our data shows that some *I. scapularis* serpins occur as exact duplicates and highly similar paralogs, a strong indicator of recent gene duplication as a means of serpin gene expansion. This has been well documented in other invertebrate serpins where tandemly arranged paralogs lead to new inhibitory functions and expression profiles [63,64,65]. Such duplicates are likely to be maintained under positive or diversifying selection. The overall conservation implies that the serpin fold and RCL remain under strong purifying constraint, while subtle sequence divergence among paralogs likely improves the target protease range, inhibitory kinetics, or regulatory control. This preserves core protease inhibitory function while generating diversity in substrate range, an evolutionary strategy that enables *I. scapularis* to maintain a flexible protease-inhibition arsenal critical for blood feeding and host immune modulation [66]. We also document that almost all but one serpins are glycosylated and contain signal peptides suggesting that nearly all *I. scapularis* serpins are secreted proteins that operate in the extracellular environment. This may help explain previous findings that serpins constitute the largest class of protease inhibitors injected into the host during feeding by *B. burgdorferi*-infected *I. scapularis* nymphs [13], underscoring their central role in modulating tick–host interactions and facilitating pathogen transmission. Glycosylation in proteins enhances protein solubility and stability, and increases their extracellular half-life enhancing serpin persistence, an important property for tick proteins secreted in saliva [25,67]. Experimental confirmation of the role of the single non-glycosylated serpin (S64c11) is needed to reveal its function and whether it is a cytosolic or regulatory serpin.

We also document that *I. scapularis* serpins occur as single and multi-exon genes which is not unique to ticks and has been observed in other invertebrates. For example, comparable mixtures of multi-exon and single-exon serpins occur in diverse taxa: the silkworm (*Bombyx mori*) encodes 34 serpin genes, of which at least five (Serpin4, 5, 7, 14 and 32) are single-exon genes [68]; the tobacco hornworm (*Manduca sexta*) has 32 serpin loci, including MsSerpin-4, 5, 7, 9 and 14, which are encoded by a single exon [69]; and in the codling moth (*Cydia pomonella*) and other Lepidoptera, most group-C serpin genes are single-exon while only a minority contain two or three exons [70]. The prevalence of single-exon genes points to retrotranspositon or duplication [71] and in this case, a projected means of serpin gene expansion in *I. scapularis*. Collectively, these characteristics indicate a system for efficient expression and deployment of serpins in salivary tissues during feeding.

The gene clustering definition we provide in this study, and the consistency of clusters across the four evaluated thresholds that we tested, further supports the prediction of local duplication as the most likely evolution process, and the maintenance of chromosomal proximity as a likely mechanism for co-regulation and rapid adaptive response. A comparable pattern of tandemly clustered serpin genes has also been reported in *A. americanum* ticks, where locally duplicated serpin families show coordinated expression and functional diversification linked to blood feeding [72]. A similar pattern of paralogous serpin clustering has been reported in *Drosophila melanogaster* and *Anopheles gambiae*, where tandem arrays promote birth-and-death evolution and diversification of protease-inhibitory functions [37,73]. This clustering is biologically meaningful to blood-feeding arthropods as expanded salivary gene families provide dosage flexibility and substrate diversity for suppressing host hemostatic and immune pathways [38,74]. Further, The enrichment of serpins on chromosome 10 points to selective retention of duplicates that enhance feeding efficiency across variable hosts and the patterns supporting local duplication are compatible with chromosome-scale “innovation zones” in which recurrent duplication can generate closely related paralogs that may later diverge, a process observed in expanded tick salivary gene families [75]. In this regard, we point out chromosome 10 as a serpin innovation hotspot, where repeated tandem duplication and diversification have produced a high-density array of functionally versatile inhibitors. This configuration mirrors *D. melanogaster* serpin clusters such as Necrotic and Spn arrays, which evolved through local duplication to regulate immune protease cascades [74,76]. The conservation of this genomic architecture across arthropods underscores a shared evolutionary strategy in which clustering enables rapid adaptation of protease-inhibitory repertoires to dynamic ecological and host-immune challenges.

In-silico prediction of serpin targets in this study is a good predictor of protease targets and will be essential in characterization studies of individual serpins, given that most serpins in *I. scapularis* have not been identified until now and very few have been studied. It is important to note that protease specificity is shaped by more than the scissile P1–P1′ pair alone. The extended RCL context (often including P2–P1 and downstream prime-side residues) can reshape recognition and therefore expand or shift target preference, helping explain why some serpins are naturally polyvalent across related proteases [77]. Beyond the RCL, exosites (secondary contact surfaces) and cofactors can strongly bias productive encounters; for example, heparin binding to antithrombin alters serpin conformation and promotes the right exosite/protease contacts, sharpening functional selectivity among coagulation proteases [78]. Glycosylation can further tune specificity by changing serpin stability, surface presentation, and cofactor affinity (including antithrombin-heparin interactions) without changing the core substrate mechanism [8]. In this study, we use the P1-P1′ criteria as a screening framework as other criteria are complex and difficult to apply consistently. Importantly, these protease target assignments illuminate vaccine and therapeutic priorities in that, anti-hemostatic serpins (inhibiting blood clotting) are attractive to block early feeding, anti-inflammatory serpins (cathepsin G, chymase, chymotrypsin inhibitors) help blunt pain and swelling that would otherwise alert the host, and tissue protection serpins (neutrophil elastase inhibitors) protect tissue and possibly dampen pathogen-induced inflammation. The multi-functional characteristic of *I. scapularis* serpins also points to complement–coagulation crosstalk, where the roles of trypsin-like complement proteases and coagulation proteases are intertwined so that serpins aimed at one cascade may yield dual benefits. From a tick-biology perspective, this genome-wide serpin atlas reveals redundancy and division of labor where multiple anti-hemostatic serpins likely guarantee clot suppression under diverse host conditions, while a panel of anti-inflammatory serpins modulates variable leukocyte mixes (neutrophils and mast cells) at different skin niches, a hallmark of blood-feeding adaptations [79]. From a pathogen transmission perspective, serpins that mute complement and neutrophil proteases may facilitate *B. burgdorferi* (and other tick pathogens) survival in the skin by reducing opsonization and protease-mediated killing, a hypothesis consistent with the general roles of these host proteases even as specific *I. scapularis* serpin–pathogen molecular links continue to be revealed by ongoing studies [25].

Gene expression analysis data points to late midgut deployment of a defined subset of serpins which stands out as the most robust and biologically inferable pattern in our data. This organization provides a strong data-supported framework for understanding how *I. scapularis* ticks manage host proteolysis during blood feeding, and also offers clear priorities for future functional and vaccine-focused studies. Based on our study, it can then be interpreted that *I. scapularis* has expanded a conserved inhibitory chassis into multiple paralogs that can be differentially deployed across developmental stages, feeding time points, and tissues. This enables the tick to maintain functional redundancy for essential processes while simultaneously exploring new inhibitory combinations that might enhance feeding success on immunologically diverse hosts. Motif analysis also supports the hypothesis of functional diversity while maintaining a conserved structure. Motif sequences are shared by majority of the serpins supporting the view that *I. scapularis* serpins form a densely interconnected network of paralogs assembled from recurrent sequence modules while containing other diversifying sequence structures that most likely define range and function. This inference is supported by the observation that the conserved motifs are found within the vicinity of the N-terminus and RCL region, supporting our earlier observation on the sequence structure that many serpins share nearly identical N-terminal RCL and RCL-core motifs, yet differ in surrounding sequence and domain context.

## 5. Conclusions

Our chromosome-resolved atlas indicates that *I. scapularis* serpins are not randomly scattered as isolated genes but instead comprise a closely organized and adaptable genomic system. Most serpins are intronless, and the predominance of secreted and glycosylated proteins is consistent with a blood-feeding ectoparasite that must rapidly produce, export, and maintain stable immunomodulatory factors in the host skin over multiple days of attachment. The uneven genomic distribution and pronounced clustering of serpin loci provide a clear window into the evolutionary forces shaping this family. In particular, chromosome 10 stands out as a serpin innovation hotspot, with densely packed tandem arrays, short intergenic spacing, and sequence relationships spanning from near-identical duplicates to substantially diverged paralogs. This architecture is compatible with recurrent local duplication processes (e.g., unequal crossing-over) and occasional rearrangements (e.g., inversions) that can expand inhibitor diversity while maintaining chromosomal proximity that may facilitate coordinated regulation.

Functionally, the combined evidence from RCL-based predictions and available experimental findings supports a practical conclusion: *I. scapularis* has evolved a flexible serpin network with the capacity to modulate multiple host pathways that threaten feeding success, including tissue-damaging proteases, coagulation, complement, and inflammatory proteases. Many serpins therefore fall at interfaces where coagulation and complement crosstalk with neutrophil- and mast cell-associated proteolysis, providing redundancy and breadth that would help maintain blood flow, limit pain and inflammation, and reduce severe local tissue disruption at the bite site. This immunomodulatory environment is also compatible with conditions that can favor pathogen establishment and dissemination, including *B. burgdorferi*, although direct causal links between individual serpins and transmission phenotypes require targeted validation. Consistent with this functional breadth, motif analysis shows that despite substantial diversification, *I. scapularis* serpins retain conserved sequence modules, particularly near the RCL, that recur in different combinations and likely reflect shared constraints tied to protease recognition and serpin stability/folding, while still permitting subfamily level specialization.

Expression profiling further supports the view that serpin deployment is programmed, not uniform: PCA and differential patterns indicate that feeding stage and tissue context dominate expression structure, whereas infection status contributes little detectable variance in this dataset. A particularly robust, testable signal is the late feeding induction in the midgut of a defined subset of basic P1 (K/R) serpins, consistent with increased protease-control demands as the blood meal expands toward engorgement. Importantly, other basic P1 serpins do not share this trajectory, supporting functional diversification within this class. In contrast, serpins with small P1 residues (S/G/A) show more gene-specific and unsynchronized changes, whereas serpins with hydrophobic/aromatic P1 residues (often associated with chymotrypsin-like targets) tend to be more broadly expressed and show modest late feeding increases across tissues rather than strict midgut restriction. Together, these genomic, motif, and expression results support a model in which *I. scapularis* serpins function as a modular, cluster-oriented innovation system that is deployed across tissues and feeding time to shape host–tick interactions at the skin and midgut interfaces, thereby promoting feeding success.

Finally, the atlas provides a practical framework for prioritizing experimental studies aimed at identifying candidates for anti-tick and pathogen-blocking strategies. In addition, experimental studies to characterize and map the functional activities of these serpins can uncover new uses for application in medicine and industry.

## Figures and Tables

**Table 1:** Chromosome-resolved catalog of all *I. scapularis* serpins, with predicted protease targets. Entries list the standardized serpin identifier, mapped chromosome, reactive center loop P1/P’ residue, predicted host protease targets, experimentally confirmed targets (when available), and a concise functional/pathway note. Predictions follow P1-guided amino acid specificity rules—R/K → trypsin-like coagulation/complement proteases (e.g., thrombin, factor Xa/XIa, kallikrein; C1r/C1s/MASP); Y/F/L/I/V/M → chymotrypsin/chymase/cathepsin G; A/S/G → neutrophil elastase/proteinase-3. RCL: reactive center loop.

Serpin	P1/P1′ Key Characteristics	Predicted Targets Based on RCL	Experimentally Confirmed Targets (Where Available)
**S1** **c1**	Basic P1 = R	Coagulation proteases (thrombin, factor Xa, factor XIa, kallikrein); trypsin-like complement	
**S2** **c1**	Small P1 = S	Neutrophil elastase; proteinase 3 (likely)	
**S3** **c1**	Basic P1 = K	Coagulation proteases (thrombin, factor Xa, factor XIa, kallikrein); trypsin-like complement (C1r/C1s/MASP)	
**S4** **c1**	Small P1 = G	Neutrophil elastase; proteinase 3 (likely)	
**S5** **c2**	Non-canonical P1 = N	Unknown specificity	
**S6** **c3**	Basic P1 = R	Coagulation proteases (thrombin, factor Xa, factor XIa, kallikrein); trypsin-like complement	
**S7** **c3**	Hydrophobic P1 = L	Neutrophil/mast-cell proteases (cathepsin G, chymase); chymotrypsin	
**S8** **c4**	Basic P1 = R	Coagulation proteases (thrombin, factor Xa, factor XIa, kallikrein); trypsin-like complement	
**S9** **c4**	Basic P1 = R	Coagulation proteases (thrombin, factor Xa, factor XIa, kallikrein); trypsin-like complement	
**S10** **c5**	Hydrophobic P1 = I	Neutrophil/mast-cell proteases (cathepsin G, chymase); chymotrypsin	
**S11** **c5**	Basic P1 = K	Coagulation proteases (thrombin, factor Xa, factor XIa, kallikrein); trypsin-like complement	
**S12** **c5**	Small P1 = S	Neutrophil elastase; proteinase 3 (likely)	Chymotrypsin; human chymase; cathepsin G [58]
**S13** **c5**	Basic P1 = R	Coagulation proteases (thrombin, factor Xa, factor XIa, kallikrein); trypsin-like complement	Trypsin IV; trypsin; FXa; FXIa; FIXaβ; plasmin; chymase; cathepsin G [58]
**S14** **c10**	Non-canonical P1 = N	Unknown specificity	
**S15** **c10**	Non-canonical P1 = N	Unknown specificity	
**S16** **c10**	Non-canonical P1 = N	Unknown specificity	
**S17** **c10**	Hydrophobic P1 = L	Neutrophil/mast-cell proteases (cathepsin G, chymase); chymotrypsin	
**S18** **c10**	Hydrophobic P1 = V + small P1′ = S	Dual: chymotrypsin/cathepsin G + neutrophil elastase (RCL favors elastase accommodation)	
**S19** **c10**	Hydrophobic P1 = L	Neutrophil/mast-cell proteases (cathepsin G, chymase); chymotrypsin	
**S20** **c10**	Small P1 = S	Neutrophil elastase; proteinase 3	
**S21** **c10**	Acidic P1 = E	Unknown specificity	
**S22** **c10**	Acidic P1 = E	Unknown specificity	
**S23** **c10**	Acidic P1 = E	Unknown specificity	
**S24** **c10**	Acidic P1 = E	Unknown specificity	
**S25** **c10**	Small P1 = S	Neutrophil elastase; proteinase 3	
**S26** **c10**	P1 = V + small P1′ = S	Dual: chymotrypsin/cathepsin G + neutrophil elastase (RCL favors elastase accommodation)	
**S27** **c10**	Small P1 = S	Neutrophil elastase; proteinase 3	
**S28** **c10**	Small P1 = G	Neutrophil elastase; proteinase 3	
**S29** **c10**	Hydrophobic P1 = I	Neutrophil/mast-cell proteases (cathepsin G, chymase); chymotrypsin	
**S30** **c10**	Basic P1 = R	Coagulation proteases (thrombin, factor Xa, factor XIa, kallikrein); trypsin-like complement	
**S31** **c10**	Basic P1 = R	Coagulation proteases (thrombin, factor Xa, factor XIa, kallikrein); trypsin-like complement	
**S32** **c10**	Aromatic P1 = Y	Neutrophil/mast-cell proteases (cathepsin G, chymase); chymotrypsin	
**S33** **c10**	Aromatic P1 = Y	Neutrophil/mast-cell proteases (cathepsin G, chymase); chymotrypsin	
**S34** **c10**	Small P1 = S	Neutrophil elastase; proteinase 3	
**S35** **c10**	Small P1 = S	Neutrophil elastase; proteinase 3	
**S36** **c10**	Small P1 = S	Neutrophil elastase; proteinase 3	
**S37** **c10**	Basic P1 = K	Coagulation proteases (thrombin, factor Xa, factor XIa, kallikrein); trypsin-like complement	
**S38** **c10**	Small P1 = A	Neutrophil elastase; proteinase 3	
**S39** **c10**	Basic P1 = K	Coagulation proteases (thrombin, factor Xa, factor XIa, kallikrein); trypsin-like complement	
**S40** **c10**	P1 = I + small P1′ = S	Dual: chymotrypsin/cathepsin G + neutrophil elastase (RCL favors elastase accommodation)	
**S41** **c10**	Aromatic P1 = Y	Neutrophil/mast-cell proteases (cathepsin G, chymase); chymotrypsin	Trypsin IV; trypsin; thrombin; human chymase; α-chymotrypsin cathepsin G [26]
**S42** **c10**	Small P1 = G	Neutrophil elastase; proteinase 3	
**S43** **c10**	Hydrophobic P1 = I (P1′ not small)	Neutrophil/mast-cell proteases (cathepsin G, chymase); chymotrypsin	
**S44** **c10**	Small P1 = G	Neutrophil elastase; proteinase 3	
**S45** **c10**	Hydrophobic P1 = L	Neutrophil/mast-cell proteases (cathepsin G, chymase); chymotrypsin	Porcine elastase; chymotrypsin; human chymase; cathepsin G [59]
**S46** **c10**	Basic P1 = K	Coagulation proteases (thrombin, factor Xa, factor XIa, kallikrein); trypsin-like complement	
**S47** **c10**	Basic P1 = R	Coagulation proteases (thrombin, factor Xa, factor XIa, kallikrein); trypsin-like complement	
**S48** **c10**	Hydrophobic P1 = L	Neutrophil/mast-cell proteases (cathepsin G, chymase); chymotrypsin	Kallikrein; chymotrypsin; human chymase; cathepsin G [59]
**S49** **c10**	Basic P1 = R	Coagulation proteases (thrombin, factor Xa, factor XIa, kallikrein); trypsin-like complement	Trypsin IV; trypsin; human chymase; cathepsin G [60]
**S50** **c10**	Acidic P1 = E	Unknown specificity	
**S51** **c10**	Basic P1 = R	Coagulation proteases (thrombin, factor Xa, factor XIa, kallikrein); trypsin-like complement	Trypsin; thrombin, cathepsin G, factor Xa [27]
**S52** **c10**	Basic P1 = R	Coagulation proteases (thrombin, factor Xa, factor XIa, kallikrein); trypsin-like complement	
**S53** **c10**	Basic P1 = R	Coagulation proteases (thrombin, factor Xa, factor XIa, kallikrein); trypsin-like complement	
**S54** **c10**	Basic P1 = R	Coagulation proteases (thrombin, factor Xa, factor XIa, kallikrein); trypsin-like complement	
**S55** **c10**	Small P1 = G	Neutrophil elastase; proteinase 3	
**S56** **c10**	Small P1 = G	Neutrophil elastase; proteinase 3	
**S57** **c10**	Small P1 = A	Neutrophil elastase; proteinase 3	
**S58** **c10**	Basic P1 = R	Coagulation proteases (thrombin, factor Xa, factor XIa, kallikrein); trypsin-like complement	
**S59** **c10**	Basic P1 = R	Coagulation proteases (thrombin, factor Xa, factor XIa, kallikrein); trypsin-like complement	
**S60** **c10**	Basic P1 = R	Coagulation proteases (thrombin, factor Xa, factor XIa, kallikrein); trypsin-like complement	
**S61** **c10**	Basic P1 = R	Coagulation proteases (thrombin, factor Xa, factor XIa, kallikrein); trypsin-like complement	Trypsin IV; trypsin; Factor Xa; Factor XIa; plasmin; cathepsin G [25]
**S62** **c10**	Small P1 = S	Neutrophil elastase; proteinase 3	
**S63** **c11**	Small P1 = G	Neutrophil elastase; proteinase 3	
**S64** **c11**	Basic P1 = R	Coagulation proteases (thrombin, factor Xa, factor XIa, kallikrein); trypsin-like complement	
**S65** **c11**	Basic P1 = R	Coagulation proteases (thrombin, factor Xa, factor XIa, kallikrein); trypsin-like complement	
**S66** **c11**	Basic P1 = R	Coagulation proteases (thrombin, factor Xa, factor XIa, kallikrein); trypsin-like complement	
**S67** **c11**	Aromatic P1 = Y	Neutrophil/mast-cell proteases (cathepsin G, chymase); chymotrypsin	
**S68** **c11**	Small P1 = A	Neutrophil elastase; proteinase 3	
**S69** **c13**	Small P1 = S	Neutrophil elastase; proteinase 3	
**S70** **j234**	Non-canonical P1 = N	Unknown specificity	

## Data Availability

The data that supports the findings will be available in NCBI’s SRA archive at https://www.ncbi.nlm.nih.gov/sra (accessed on 10 February 2026) following an embargo from the date of publication to allow for commercialization of research findings.

## Supplementary Materials

The following supporting information can be downloaded at: https://www.mdpi.com/article/10.3390/genes17040361/s1, Supplementary Figure S1. Temporal expression of significantly expressed serpins. Supplementary Table S1. Serpin loci and genomic coordinates. Supplementary Table S2: Protein sequences of all the 74 *I. scapularis* serpins. Supplementary Table S3. Pairwise similarity and paralog classifications. Supplementary Table S4. Signal peptides, molecular weight, isoelectric point, and glycosylation motifs. Supplementary Table S5. Serpin cluster definitions and orientation. Supplementary Table S6. Quantitative gene expression statistics of serpin deployment vs infection. Supplementary Table S7. Motif occurrences by serpin.
