# Supplementary material for: Chromosome-Scale Atlas of Ixodes scapularis Serine Protease Inhibitors
Source: Genes (Basel). 2026 Mar 24;17(4):361. doi: 10.3390/genes17040361 (PMC13116602; doi:10.3390/genes17040361)
Supplement: Supplementary file 1 [file genes-17-00361-s001.zip › Supplementary Figure S1.pdf]

Supplementary Figure S1: Temporal expression of significantly expressed serpins.

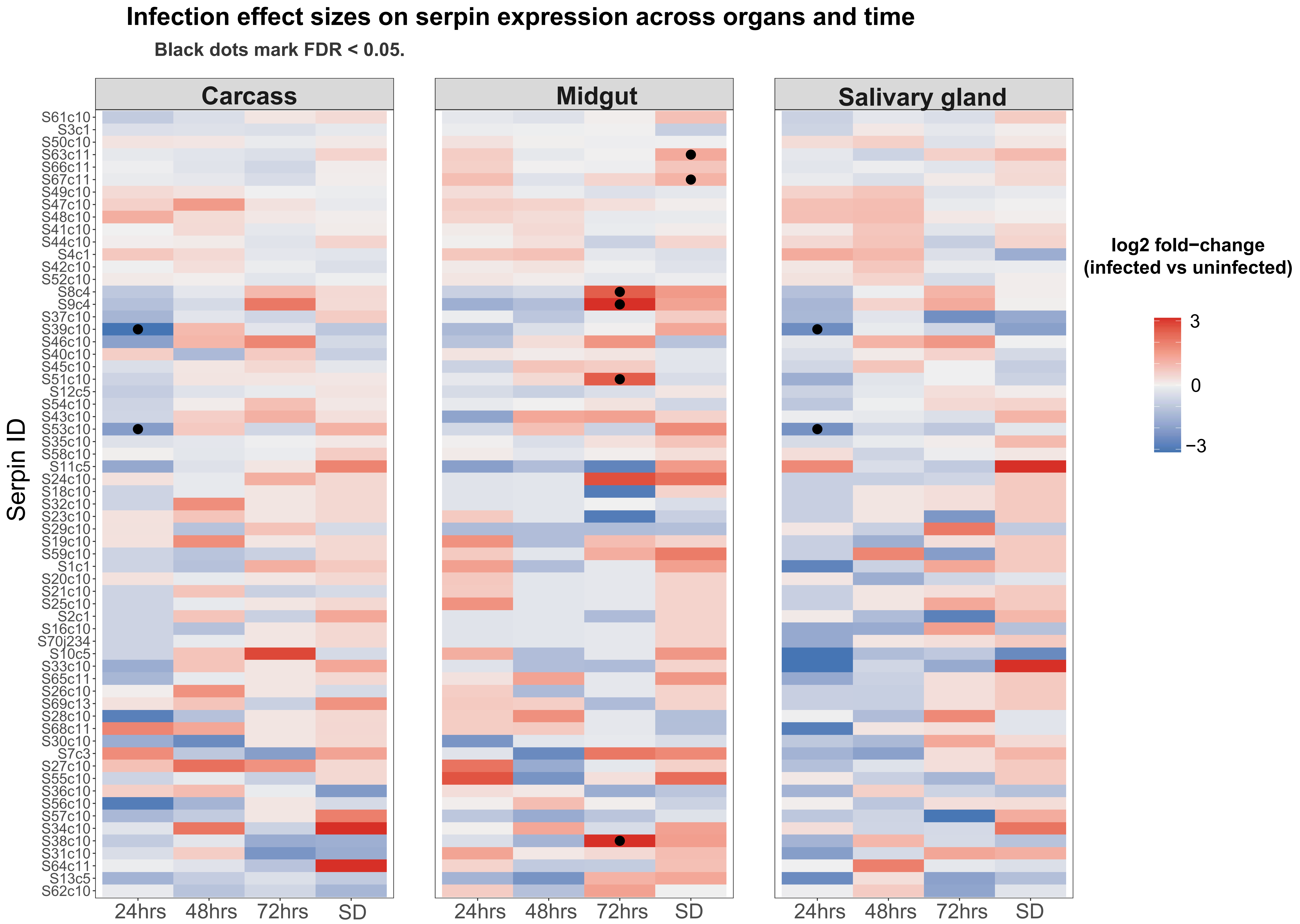

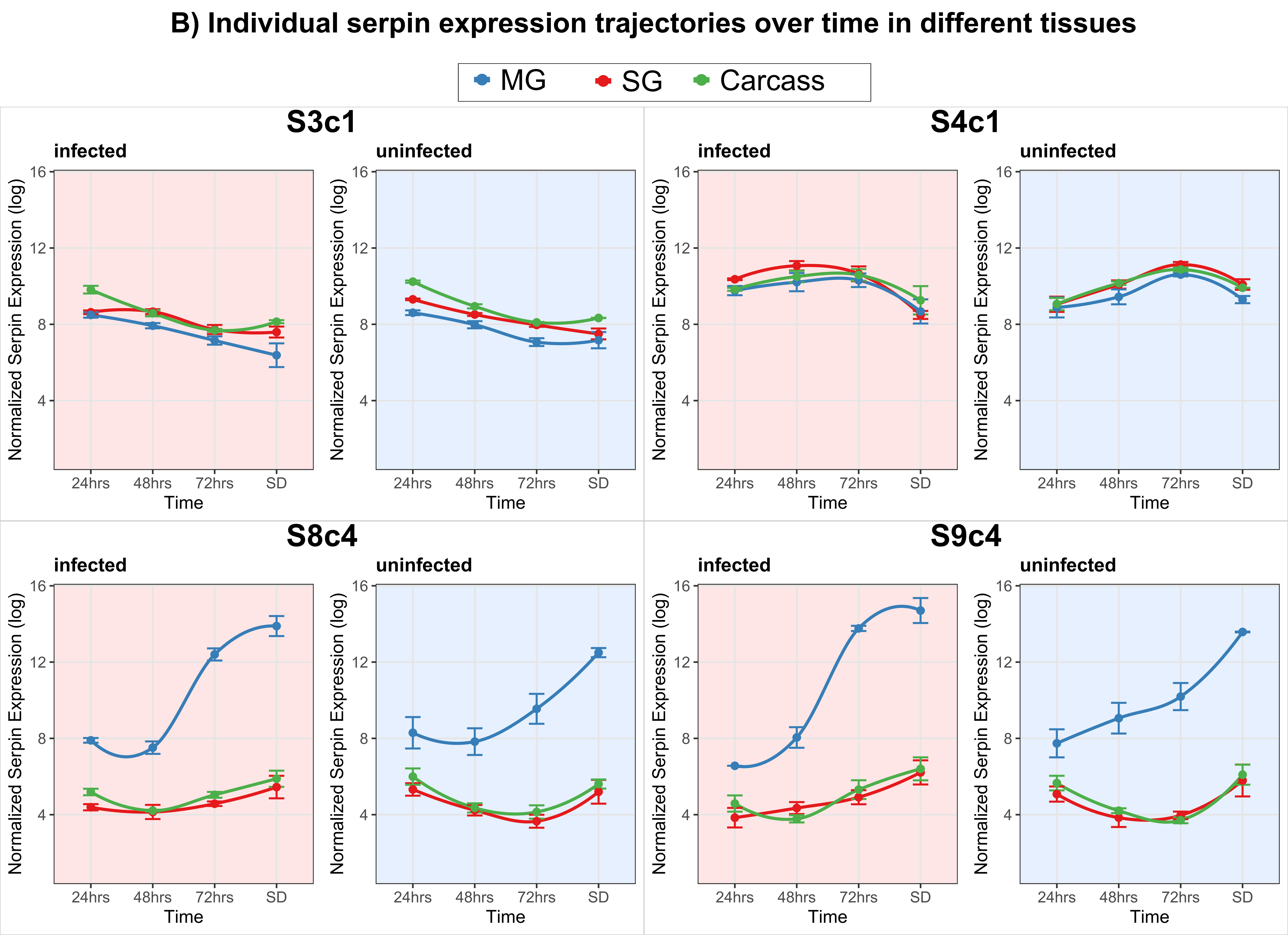
B) Individual serpin expression trajectories over time in different tissues
MG
SG
Carcass
S3c1
infected
uninfected
S4c1
infected
uninfected
S8c4
infected
uninfected
S9c4
infected
uninfected
Normalized Serpin Expression (log)
16
12
8
4
24hrs
48hrs
72hrs
SD
Time

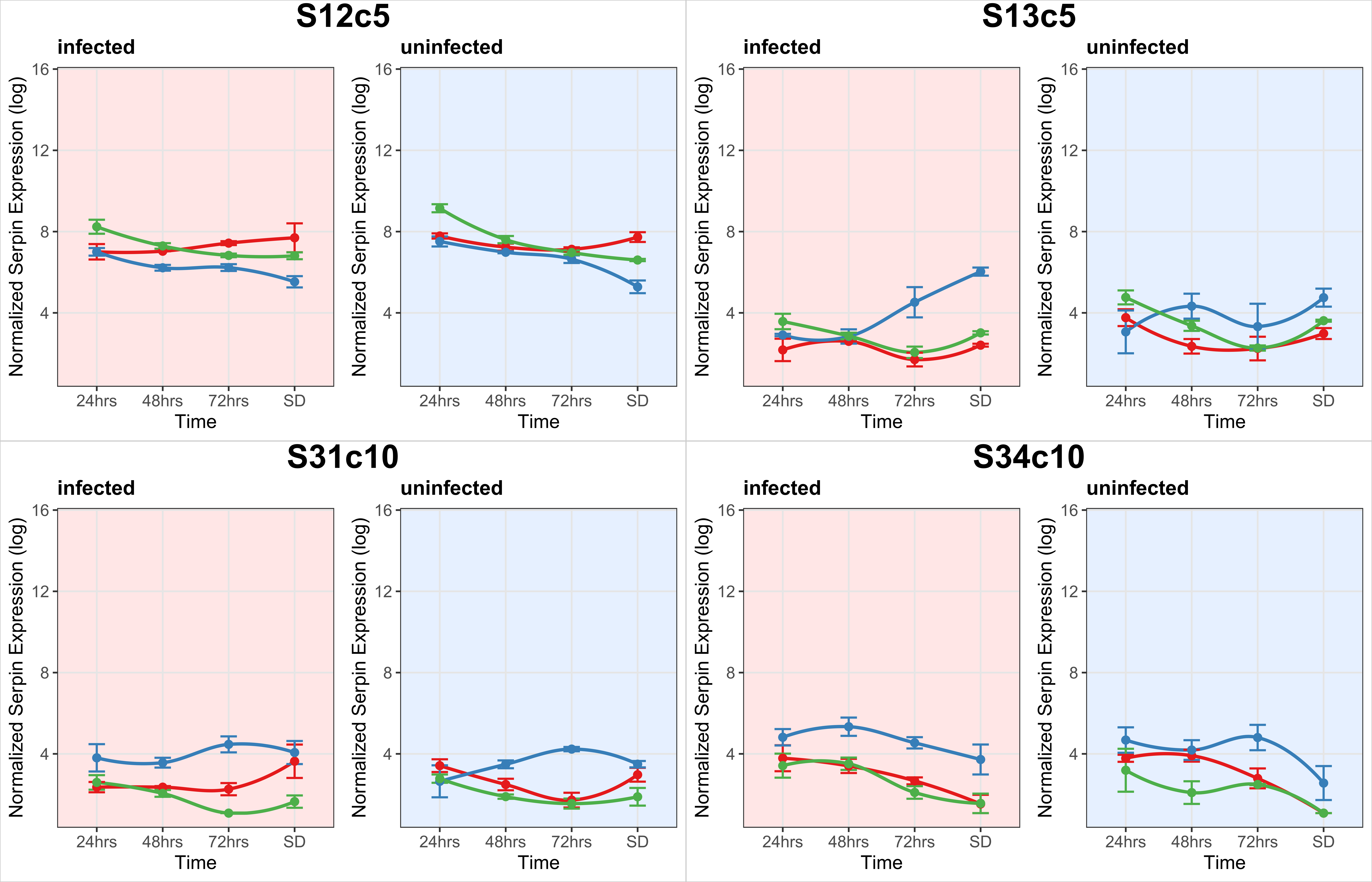
S12c5
infected
uninfected
S13c5
infected
uninfected
S31c10
infected
uninfected
S34c10
infected
uninfected
Normalized Serpin Expression (log)
16
12
8
4
24hrs
48hrs
72hrs
SD
Time

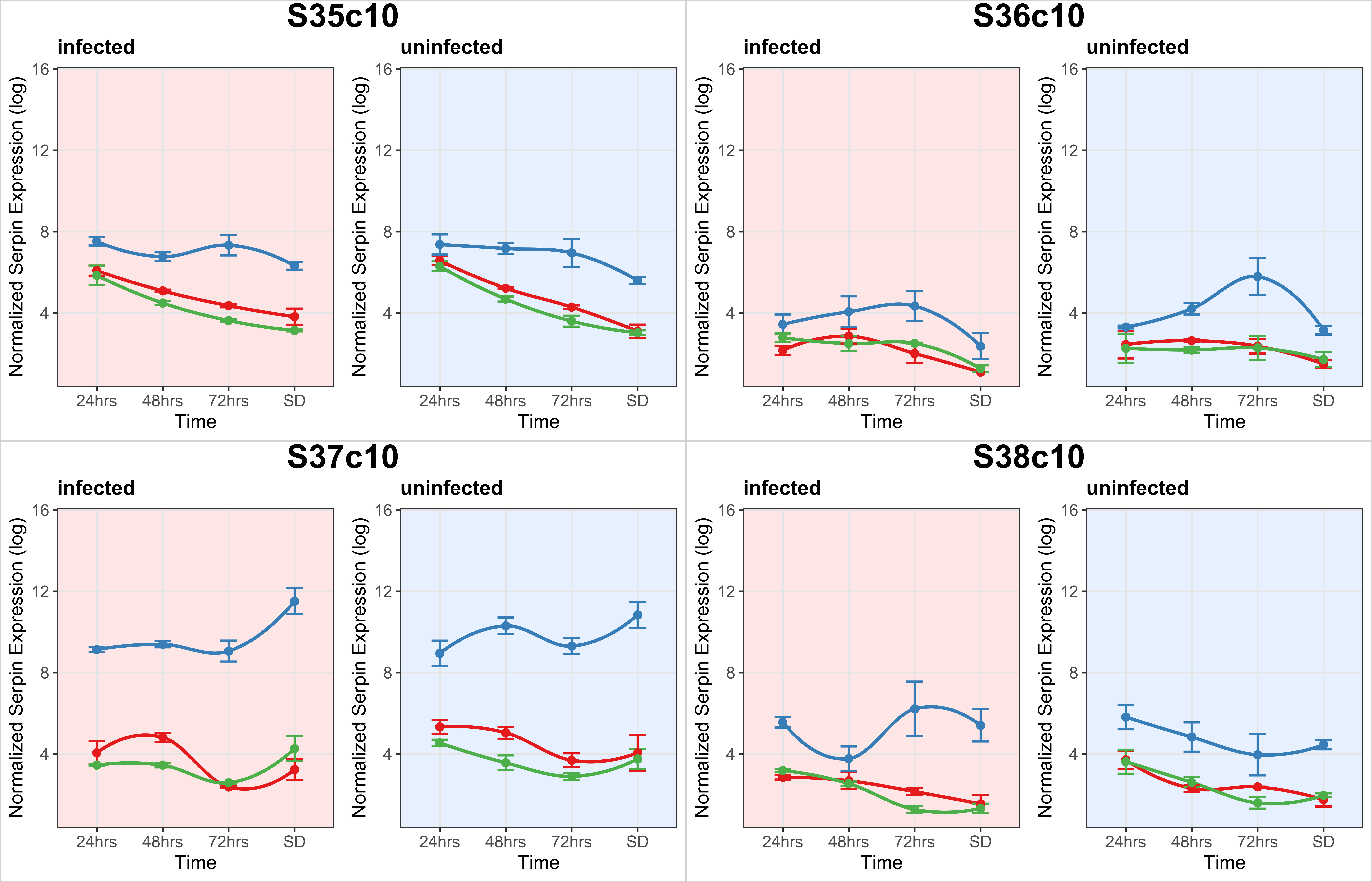
S35c10
infected
uninfected
S36c10
infected
uninfected
S37c10
infected
uninfected
S38c10
infected
uninfected
Normalized Serpin Expression (log)
16
12
8
4
24hrs
48hrs
72hrs
SD
Time

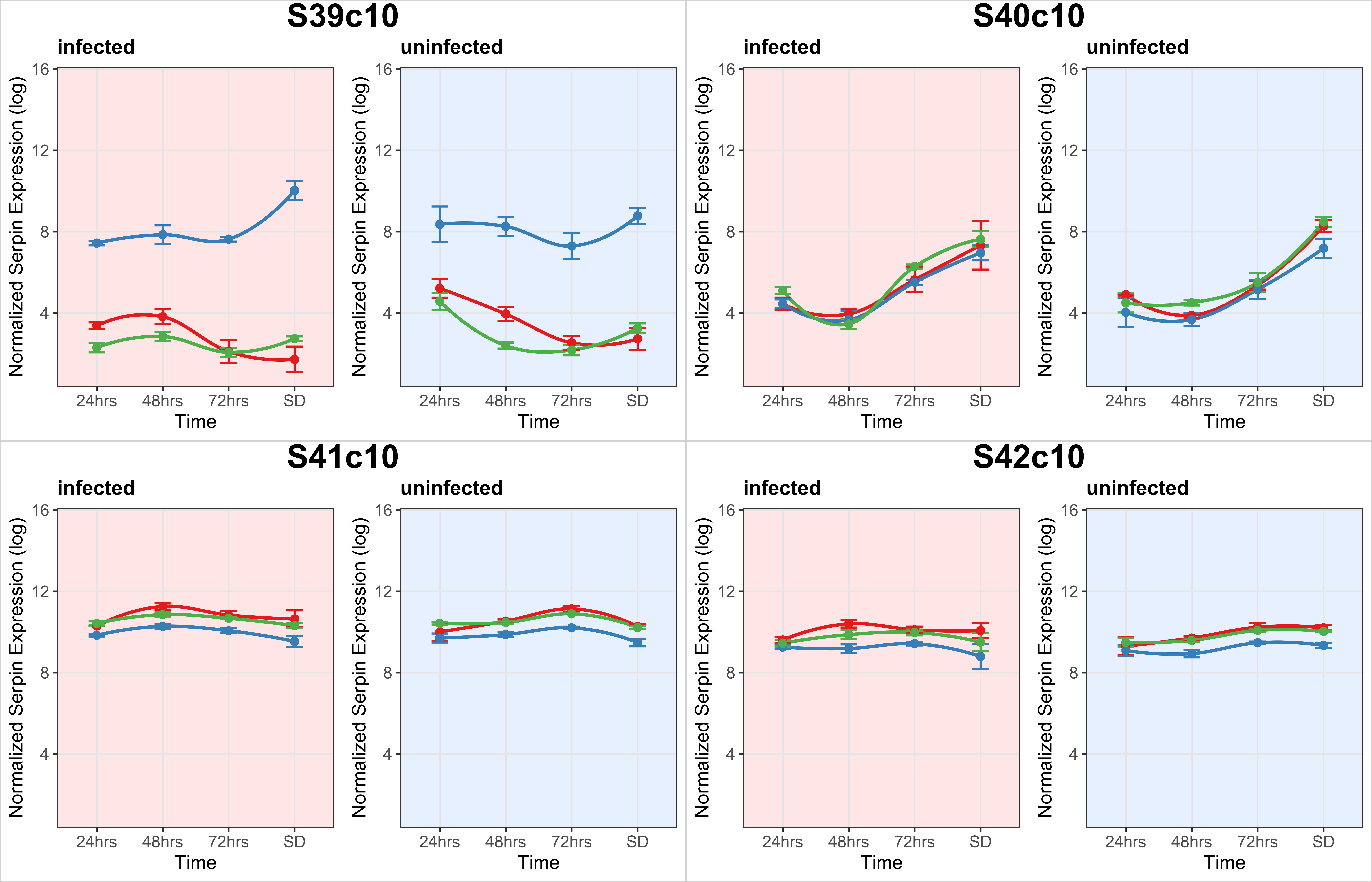
S39c10
infected
uninfected
S40c10
infected
uninfected
S41c10
infected
uninfected
S42c10
infected
uninfected
Normalized Serpin Expression (log)
16
12
8
4
24hrs
48hrs
72hrs
SD
Time

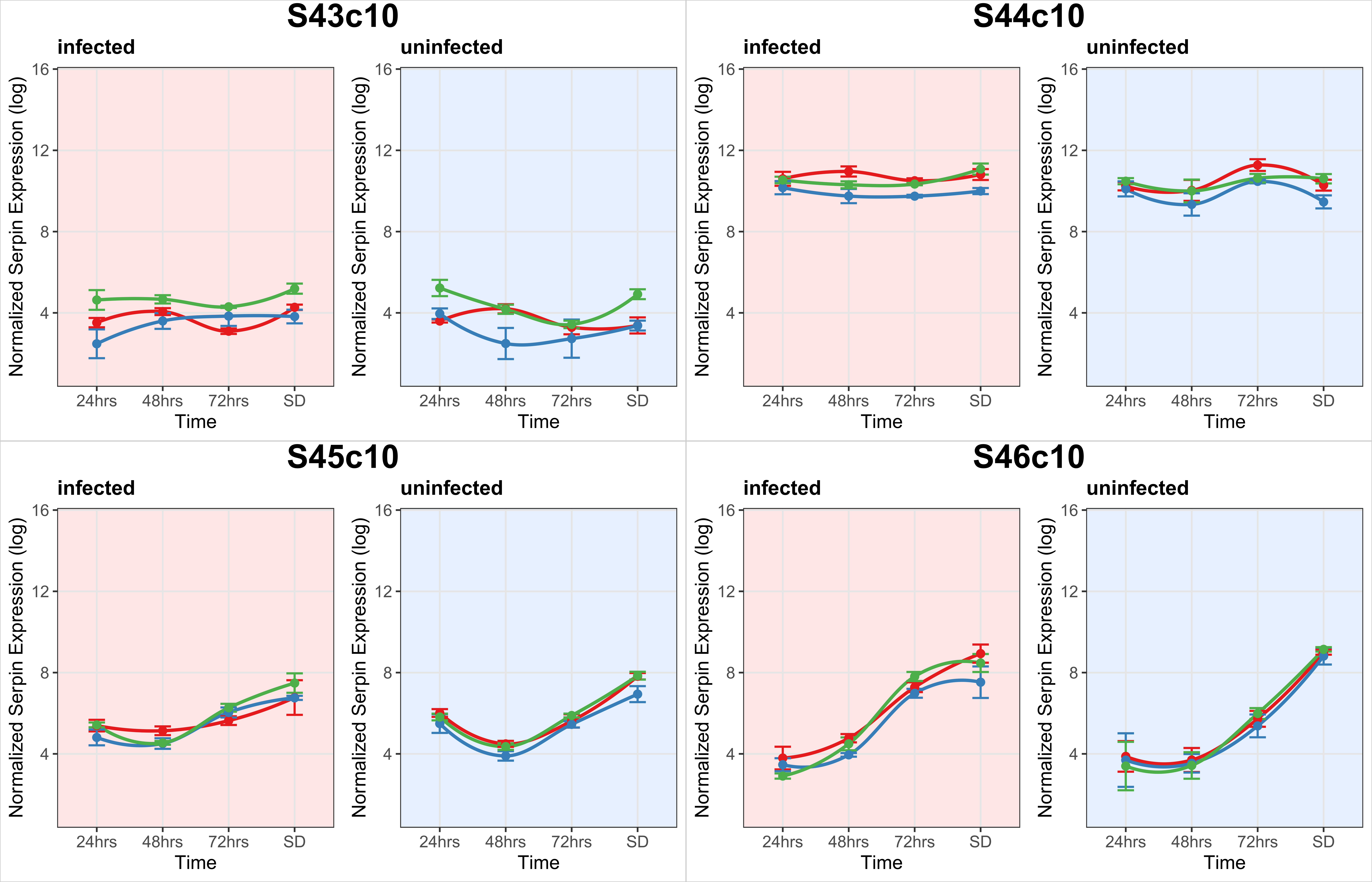
S43c10
infected
uninfected
S44c10
infected
uninfected
S45c10
infected
uninfected
S46c10
infected
uninfected
Normalized Serpin Expression (log)
16
12
8
4
24hrs
48hrs
72hrs
SD
Time

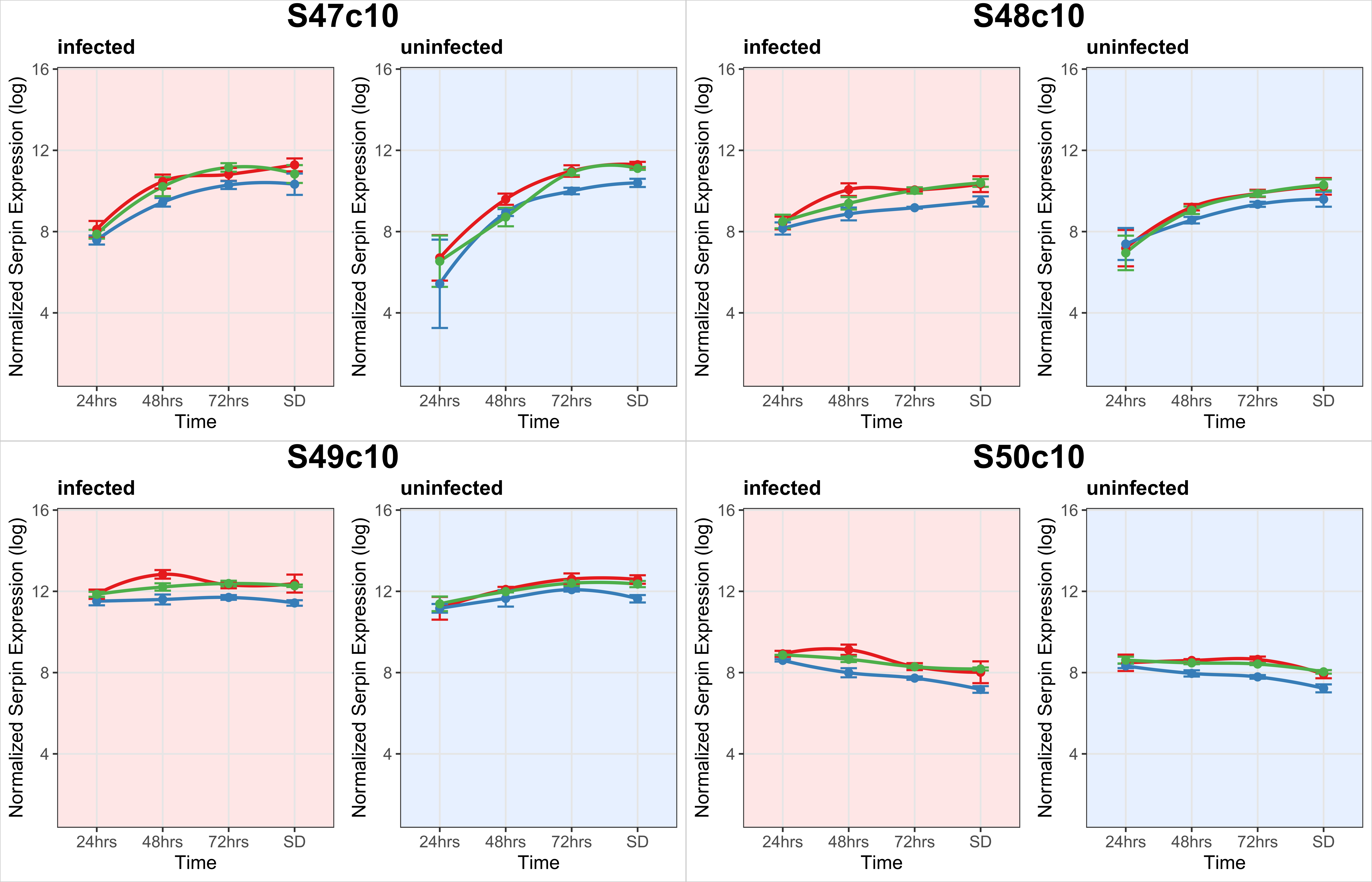
S47c10
infected
uninfected
S48c10
infected
uninfected
S49c10
infected
uninfected
S50c10
infected
uninfected
Normalized Serpin Expression (log)
16
12
8
4
24hrs
48hrs
72hrs
SD
Time

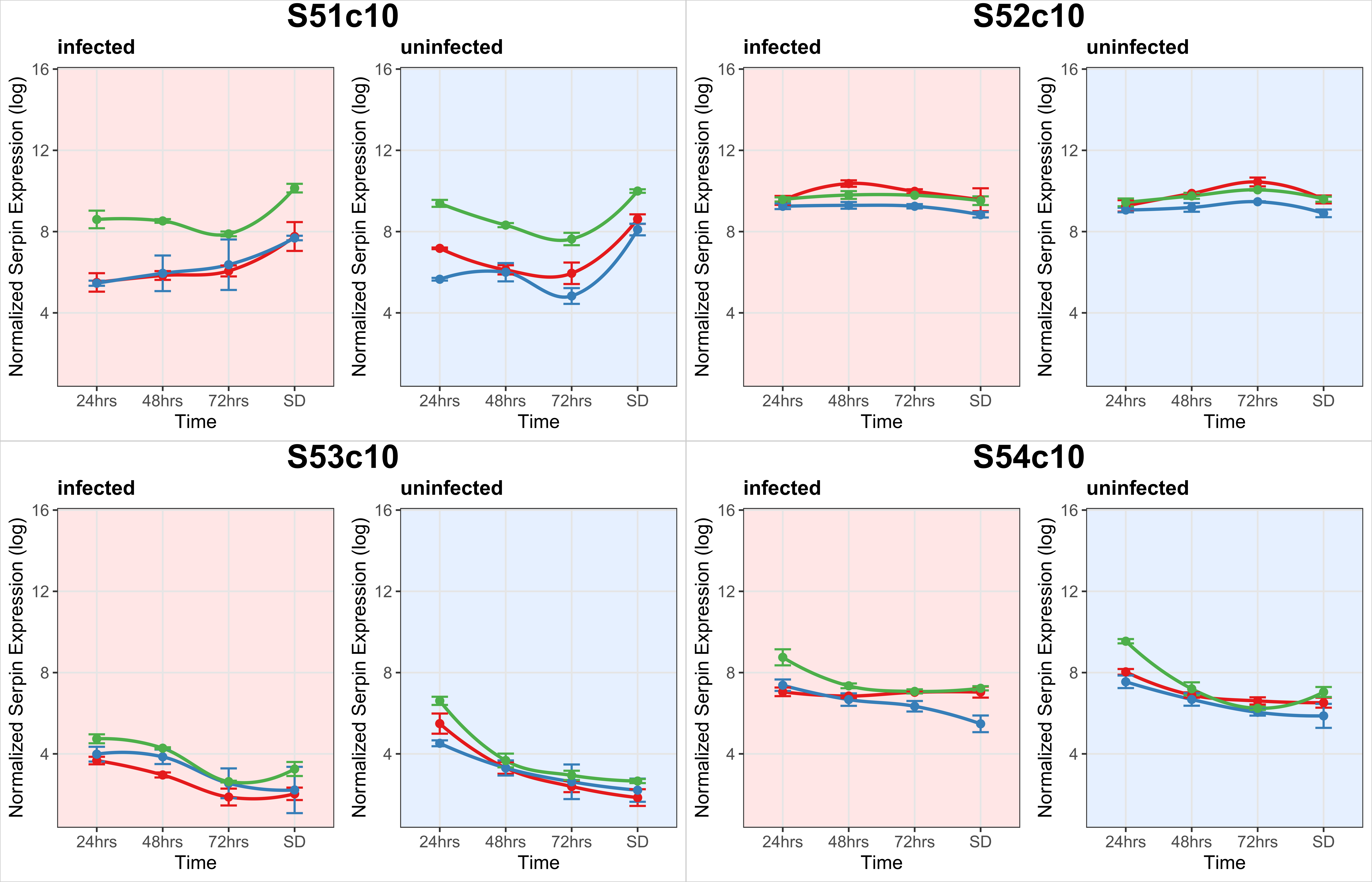

S51c10
infected
uninfected
S52c10
infected
uninfected
S53c10
infected
uninfected
S54c10
infected
uninfected
Normalized Serpin Expression (log)
16
12
8
4
24hrs
48hrs
72hrs
SD
Time

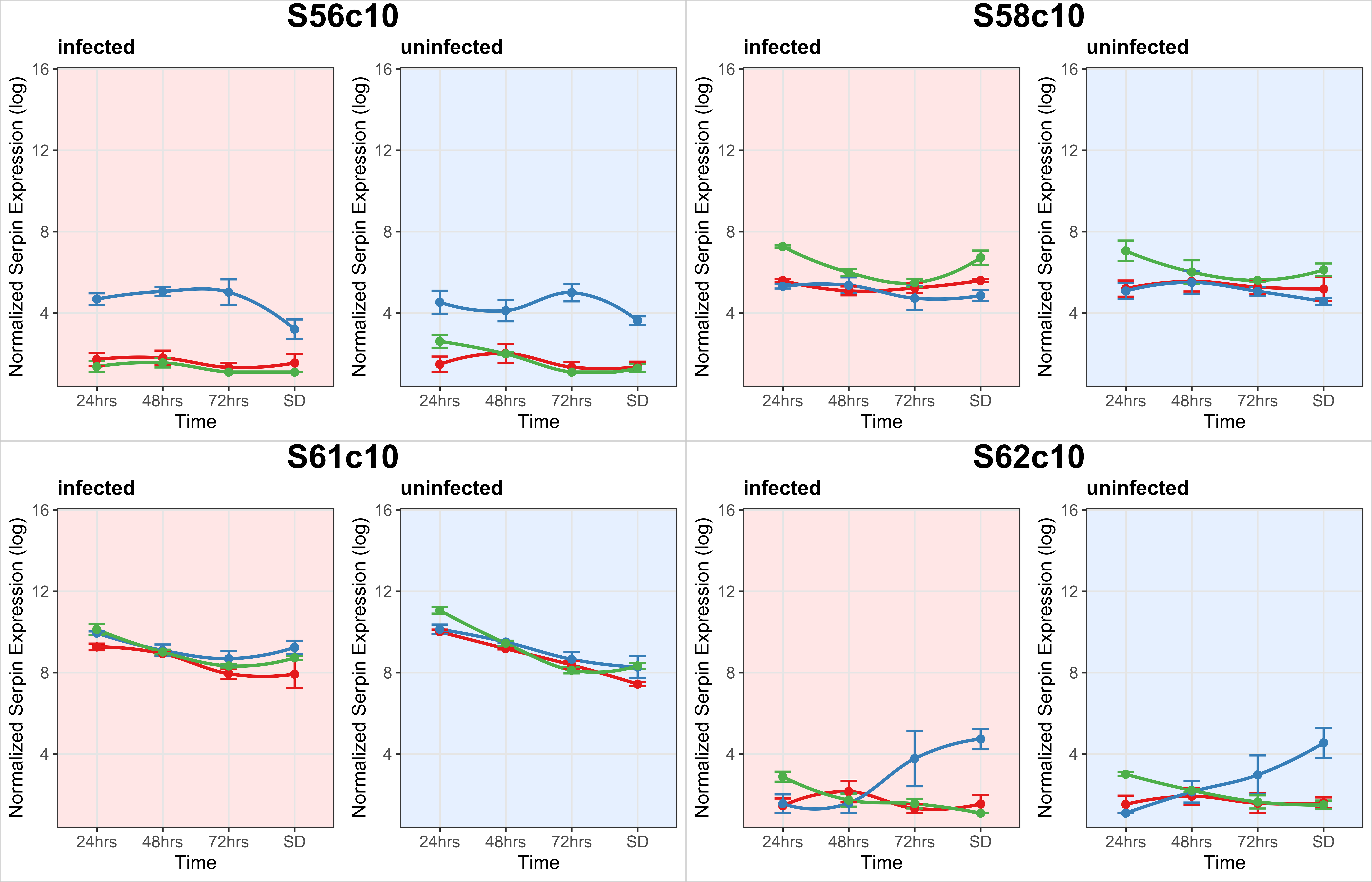
S56c10
infected
uninfected
S58c10
infected
uninfected
S61c10
infected
uninfected
S62c10
infected
uninfected
Normalized Serpin Expression (log)
16
12
8
4
24hrs
48hrs
72hrs
SD
Time

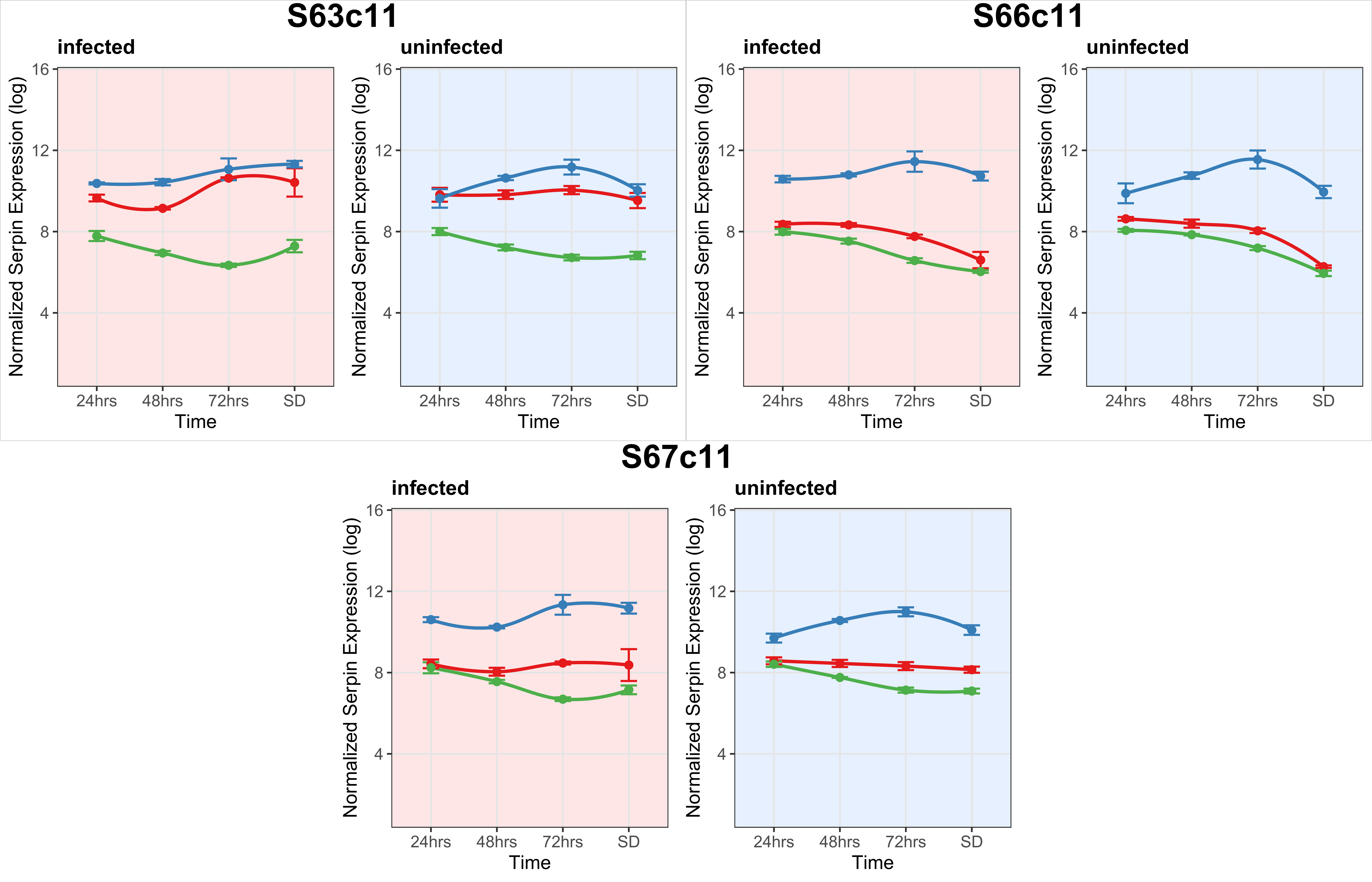
S63c11
infected
uninfected
S66c11
infected
uninfected
S67c11
infected
uninfected
Normalized Serpin Expression (log)
16
12
8
4
24hrs
48hrs
72hrs
SD
Time
