# Supplementary material for: Chromosome-Scale Atlas of Ixodes scapularis Serine Protease Inhibitors
Source: Genes (Basel). 2026 Mar 24;17(4):361. doi: 10.3390/genes17040361 (PMC13116602; doi:10.3390/genes17040361)
Supplement: Supplementary file 1 [file genes-17-00361-s001.zip › Supplementary Table S2.pdf]

## Supplementary Table S2: Protein sequences of all the 74 *Ixodes scapularis* serpins

Sequences were translated from genome exons (denoted with gXXX.t1/t2) and sequence manually confirmed by NCBI BlastP. Annotation is based on the best NCBI BlastP hit. The reactive center loop (RCL) sequences are highlighted in yellow while the conserved N-terminal motifs are highlighted in green. For Two exon genes, residues of the second exon are underlined.

---

>s1c1_g1610.t1_Chr1 XP_029832862.2 (PI=97.3) intracellular coagulation inhibitor 3-like [Ixodes scapularis]
MMLAEKVFLILFVLLAGDSECCTEDSDMKLVRAQNHFALKLLKELSSEAPESNIFFSPTSISVALAMVYAGA
RGKSEAELSTALGHTAAGLSSRESILASYKTILAQQQTDDNVSVMIANAVFVQKNLKVLESYQKELVDTFAA
MFRSVDVGTEKSAMESEVNQWVKNKTRGKISGFKIPANTIMALLNAIYFKGLWETPFHVNDTFPLNFYNK
RSKKIKVEFMQRRGNVPISFGPNYAAIELSYKGDKHGMVIVFPYKIIGLPKLLDAMTLETIERIQGSLKSEAV
WVRLPKFDLKTEYGLISALKKLGVRSIFSHADLSGITGDRGLWVTEVQHKAAIEVNEEGMGVSAPMKLEN
EGRFPTPFPVNSPFLFYIHEKATGRVLFLGAVHELPVAKVKPALR

>s2c1_g1612.t1_Chr1 XP_040063232.1 (PI=84.7) intracellular coagulation inhibitor 3-like [Ixodes scapularis]
AEDSDMKLAQAQNHFALKLLKELSSEAPESNLFFSPTSISVALAMVYAGARGKSEAELSSALGHTAAGLSS
RESVLESYKRILAQQQTDDNVSLMIANAVFVQKNLKVLESYQKELVDTFAAMFRSVDVGAKKSDMESEVN
EWVKNKTRGKIAGFKIPANIVMALLNAIYFKGLWKTPFKLNHTSSLPFYNKGSQEIKVETMTREGKVPFTYK
PDFAAIELPYKGDRHCMVIVLPHEKKGLPKLQDAITVKSIEKIQGSLHYETVKIQLPKFNLKTKYGLIPVLKKL
GVRSIFSDADLSGITGDRGLRVTEVQHKAAIEVNEEGTVASGATELGGGRSLPTPFFVNRPFLFYIREKAT
GRVLFLGQVHELPAAKPTIG

>s3c1_g5467.t1_Chr1_Serpin26 XP_040071239.2 (PI=99.5) leukocyte elastase inhibitor-like [Ixodes scapularis]
MKLLLPLLWCFFVPMGVHSIHFRRKTARLQQQHAAAINSVVESINSLGIRLLQTAGDRNMLLSPLSLNVVLN
MVLLGARGRTAYQMSSQLNHPDKKGLSELLKDMTSTGGNKRATSLDMASAMLIEQGAPYNESYHREIED
LFDANLGVTQFAQNPDEIVKEVNAWANHKTRGRIDKFLQQAPDAATKMLVLNAMYFKGNWKTQFDPEFT
EKRTFHNLDGTKSSVPMMFLIHGFELGYDAALDVDVLKLPYADDHFSMILVVPRNRQNSIGAVVQGLSTSK
LNSMLRNLDTQNVELTMPKLNVDQLLKLRGSLEQLGLKVPFSESANFSGISEKLDLRLNEVMHKAALDVD
EEGTRAVAATQAQFVSKSLVQFTQFTVDHPFLAFIRHEKSGAIVFLAHIVSMDA

>s4c1_g12634.t2_Chr1_Serpin42-like XP_040356992.2 (PI=100) intracellular coagulation inhibitor 1 [Ixodes scapularis]
MMLGRLAALLVLVVSCKAQEELKLTLANNRFGLRLFHALPSAADVNVFFSPYSVSTGMGMTYAGARRDTA
EELFRGLGYSASGLTAPQVSDLYARHTQRLLSSESQSTLKVANGVAIQENLTLLDSFRNTLESSFNAEVHQ
VDFVHRKQDAFELLNRWVKQKTSDKIDKLFDEPLNSSTRLVLLNAIYFKGVWNTQFRRVFTQKRQFWNG
GVSPTLVDTMMQNMHVGYGSFGDLDVDVAELPYRGRDYSMVILLPKRNDGVDRLKGNLTVEIMRSLLSE
LRERDVDIFLPKFKLESTYQLKGPLTALGIRQIFSNGADLSGISDRNLRVSAVVHKAVLEVSEEGTEASAAT
GILAPLGSGMPVEFVADHPFLLFIRNTRTNDIIFAGQVNKL

>s5c2_g9658.t1_Chr2 XP_040067801.1 (PI=94.7) intracellular coagulation inhibitor 2-like [Ixodes scapularis]
MLAQNVLIVLFVLLTGDGECNAEDSDMKLARAQNHFALKLLKELCSEEPESNIFFSPTSISVALAMVYAAARGKSEAEFSTALGHTAAGLPSRESTLESYKKILAEQQTDDNLSLMIANAVFVQKKLKVLKSYRKELVDIFAAMYRSVDVGGGKSAMESEVNQWVKNKTKDQISGFKIPAGTITALLNAINFKGLWETPSDLKYSFSLPFYNKGSEVVMAETMSRDGTVLFTSEPGLKCEAIEYPYKGGRHSMIFVFPNEKNGLAELRQAITVESIEKIQRNLKETVLMFQLPKFDLKTEYNLVPALKKLGVGSIFSDADLSGIPGGEGQVVTEVQHKAAIEINEEGTVAAAATAVRASKNRTQSFVVNRPFLFYICEKATGRLLFLGEVHALPAIK

>s5c2_g9659.t1_Chr2 XP_040067801.1 (PI=94.7) intracellular coagulation inhibitor 2-like [Ixodes scapularis]
MLAQNVLIVLFVLLTGDGECNAEDSDMKLARAQNHFALKLLKELCSEEPESNIFFSPTSISVALAMVYAAARGKSEAEFSTALGHTAAGLPSRESTLESYKKILAEQQTDDNLSLMIANAVFVQKKLKVLKSYRKELVDIFAAMYRSVDVGGGKSAMESEVNQWVKNKTKDQISGFKIPAGTITALLNAINFKGLWETPSDLKYSFSLPFYNKGSEVVMAETMSRDGTVLFTSEPGLKCEAIEYPYKGGRHSMIFVFPNEKNGLAELRQAITVESIEKIQRNLKETVLMFQLPKFDLKTEYNLVPALKKLGVGSIFSDADLSGIPGGEGQVVTEVQHKAAIEINEEGTVAAAATAVRASKNRTQSFVVNRPFLFYICEKATGRLLFLGEVHALPAIK

>s6c3_g139.t2_Chr3_Serpin25-like XP_040070776.1 (PI=96.0) intracellular coagulation inhibitor 2-like [Ixodes scapularis]
MLAEKVFLVLFVLLAGNRRCHAEDSDMKLARAQNLFSLQLLKKLSSEKPESNIFFSPTSISVALAMVYAGARGKSETEISTALGHTAAGLSSRESILESYKKILAEQQTDDNVSLMIANAVFVKKTLNVLESYQKEPVDIFAAMFRPVDVGAGNSSMELEVNEWLKNKTRGKISGFKIPPNTIMALLNDIYFKGAMEHSFDPNDTCILSFHNKGFEVVMVETMTRLAKVPFTSEPEFEAIELSYKGDQHCMVIILPSEKKELPKLRDAMTVESIKQIQKSLKDETVKIQLPKFDLKTEYGLVPAVKKMGVRSVFSDADLSRVTGDGVLRVTEVQHKAAIEVNEEGTVAAGATVVVIEKRAPHSFALDRPFFFYIREKATGRMLFLGEVHALPAAKPTLG

>s7c3_g14695.t1_Chr3_Serpin43 XP_042148092.1 (PI=99.7) leukocyte elastase inhibitor-like [Ixodes scapularis]
MFRSGTAKEVSPLASDFLFTFSVNLYKQLQAEEGTNSNIVCSPFSIAAALSMINAGARNNTQKQISHLLHVKDPDVHDKFCVFFDRFCFLPPEMMLFIASRIYASFEFSPLEDFACLLKKIYKSSMKGIEFRGDPNVSRCQVNSWVAETTKFMIRNCLPLNAFQKTTQVVLANAVYFRGFWDSPFRLDAASRVAFHEDATTTKAVDMMFRRGTFMMCYSDELKVTALKIPYRGGKMSLVVLFPEKVDGLTNLVKQLTPYKLTRLLRDVTRPMRIELRLPKFKLEHTIDLKETLSVMGATDLFTERADLSGMSVPPCPLFSSAIHKCVLDVNEEGDETAADAGMEGVSLTLDHPFMFLVEHHDPVAIILMGVLRKI

>s8c4_g22717.t1_Chr4_Serpin21 XP_002402925.4 (PI=97.3) intracellular coagulation inhibitor 1 [Ixodes scapularis]
MFAPTALFFLMAVASCYSQSDEQNLAKANNQFSVSLLKQLIAEKPDGNVFFSPTSISTALAMIYAGAGGSSEEELSTVLGQAGFGLTDRDAVLAAYKKLLAGTNSRKVTLDIANSVLIEKRLPVLDSFKKALTESFGAELRSVDFANDGPRVQSEVNKWVSQKTRGKIPSIVDDGFPMNTVMFLLNAVYFNGTWHNQFPQKNTVPRPFFNRGSEEVHVKTMALRGSIRHTRLDDLKAQAVELPYQGEEYSMVIVLPNAKMGLSQLREGLTVSMLQKITNQMSAKTVSLTLPKFELQTNYDLIPTLKQMGLKSVFDEKSDFSGITGDKSLYVSGVRHKAMVEVNEKGTVAAAVTSISVAVRTSLIRPKIVHFHVDRPFLFYIRNRATGRLLFMGEVHQLQA

>s9c4_g22717.t2_Chr4_Serpin12-like XP_029851414.3 (PI=100) intracellular coagulation inhibitor 1 [Ixodes scapularis]
MFAPTVLFLLMAVASCYSQSDEQNLAEANNEFSVSLLKQLTSAKPDNNVFFSPTSISTALAMIYAGAGGSS
EEELSKVLGLAGFGLTDRDAVLAAYKKLLAGTNSGKVTLDIANSVLIEKRLPVLDSFKKALTESFGAELRSV
DFANDGQRVKSEVNKWVSQKTRGKIPSIVDDGFPQNTVMFLLNAVYFNGTWHNQFPQKNSVPRPFFNR
GSEEVHVMTMALRGSIRHTRLDDLKAQAVELPYQGEEYSMVIVLPNAKMGLSQLREGLAVSRLQKITNQM
SAKTVSLTLPKFELQTNYDLIPTLKQMRLKSVFDEKSDFSGITGDKSLYVSGVRHKAMVEVNEKGTVAAAV
TSISMGMRSSLGPKIVDFHVDRPFLFYIRNRATGRLLFMGEVHQLQA

>s10c5_g30598.t1_Chr5_Serpin29-like XP_040078043.1 (PI=93.3) intracellular coagulation inhibitor 3-like [Ixodes scapularis]
MMLAEKVFFVVFVLLGADSKGNSEDSDMKLAQAQNHFALKLLKELCTENQESNIFFSPASISVALAMVYAG
ARGKSETELSAALGHTAAGLSSRKSILESYKKILAEQQTDDNVSLMIANAVFVEKTLKVLESYQKELVDTFA
AMFRSVDVGAEKSDMEAEVNEWVKNKTRGKISGFEIPADTVMALLNAIYFKGLWKTPFEPKYTSPLPFYN
KGSEEVKVETMTRTSMVPITFEPDFEAIELSYEGDGHCMVIILPRNHTSKALAELRDSMTVESIEKIKESLK
RESVTIQLPKFDLKTEYGLIPALKKLGVRSIFSDADLSGITGDGGLRVTDVQHKAAIEVNEEGTVAGAATDV
VLGRILPARFFVNRPFLFYIREKATGRVLFLGEVHELPAAKVKPALRQCDSDPTYGDGIVFQIQSRMQ

>s11c5_g32844.t1_Chr5_Serpin16-like XP_029843626.2 (PI=97.2) serine protease inhibitor-like [Ixodes scapularis]
MLQTRTTFFLVTIAACVVGSTGESRWQNAEFSLNLLRELPRNKSTNTVLSPIGAFIPLAILNSGANGETGRE
IQRVLSSTKTYSREDLLSSFQAFYETVYHRDANNSYLEQANVFVVKKETKKKLRPAFRKLLDKGNVLHAS
MWEMSGQLPFQIDQWVGAETNKKITHILDADALDQNTVMLAINVIRFKGLWSSKFEQRLNSIDTFHNADG
TAVPTTFMFKRFKTGYHFDEDLKTHVVALPYQKLRARFIIFLPLEPARLDDLKSKLSATRWRSTLQRLDKRN
VALSLPKFKLSSRYDLVGPLKKLGINILFGRGADLSGIDGDRDLFVSGFLQVSKLQVDEAGSEAESVTVVK
VSGKSAETAATVTVDHPFLFFITAGADDVVLFAGQVNHIEKA

>s12c5_g34013.t1_Chr5_Serpin20-like XP_040071636.1 (PI=100) intracellular coagulation inhibitor 1 [Ixodes scapularis]
MKRCTLVALLLAVAAARADRDAVSRASNDLGLALYRKLAEVDSKGGNVFLSPVSVAAILGMVQLGARGRT
RQELDEALAGGSNKLGKVSGDDLAAGFGQLLKDLRAEKGYDLRIASAVFIASGLPIFERYKKDLDKHFASG
IYSADFAGNGREAAQDVNNWVRDMTEDRIPEILERPLPPSAPLLVLNAVYFRGLWLNPFKPNETRKEDFY
NRGTRAVPVDMMHVKEELVYAYSDALDADILELPYEGDRVVMVLVLPRKRDGLPDLERRFALEPIREALAG
AVQRRVDVRLPKFQLQLSYSLRSVLQSLGVREAFNAQGANLSGISGSRRLSLDEVLHKALLDVDEQGTEA
VALSSGIVRHSRPPEEEVQFKADHPFIFFIEDVRSQTLLFLGRLQEV

>s13c5_g34014.t1_Chr5_Serpin19 XP_029834128.2 (PI=99.7) leukocyte elastase inhibitor A [Ixodes scapularis]
MWFPALLALACSCCVIAEPNELEDPSYGGVVRASNDFGLTLYTTLANGSDGNLFFSPWSLSRVLTMVLLG
ARNETASELSRALSLDSIGDADVALAAQRRQAQQLGKSDELATTSMALTRVGDPVSAEYLASLDRYLDDG
GVMQVDFSRADELLNRVNGEVSRLTGGRIRDALRRSPDPLSKLLLLNAVHFKGVWAKAFSPNDTFDGVF
RGATKNTPVRMMAAKGKFRLSYDGAAYVLELPYTGDSSLVIVLPRNRLQRDLQDLEHRLQELLMAPPAEL
RSLELELPQLSLHSSLDLKDALQQMGVRALFAEHEANLSGMQPDGGLFVEDVHHEAALDVDEKGTQASA
STVAVIVSRIGTPRFSVDRPFVFALRHGPSGLLLFVGRVLDL

>s12c5_g34013.t2_Chr5_Serpin20-like XP_040071636.1 (PI=100) intracellular coagulation inhibitor 1 [Ixodes scapularis]
MKRCTLVALLLAVAAARADRDAVSRASNDLGLALYRKLAEVDSKGGNVFLSPVSVAAILGMVQLGARGRT
RQELDEALAGGSNKLGKVSGDDLAAGFGQLLKDLRAEKGYDLRIASAVFIASGLPIFERYKKDLDKHFASG
IYSADFAGNGREAAQDVNNWVRDMTEDRIPEILERPLPPSAPLLVLNAVYFRGLWLNPFKPNETRKEDFY
NRGTRAVPVDMMHVKEELVYAYSDALDADILELPYEGDRVVMVLVLPRKRDGLPDLERRFALEPIREALAG
AVQRRVDVRLPKFQLQLSYSLRSVLQSLGVREAFNAQGANLSGISGSRRLSLDEVLHKALLDVDEQGTEA
VALSSGIVRHSRPPEEEVQFKADHPFIFFIEDVRSQTLLFLGRLQEV

>s13c5_g34019.t1_Chr5_Serpin19 XP_029834128.2 (PI=99.7) leukocyte elastase inhibitor A [Ixodes scapularis]
MWFPALLALACSCCVIAEPNELEDPSYGGVVRASNDFGLTLYTTLANGSDGNLFFSPWSLSRVLTMVLLG
ARNETASELSRALSLDSIGDADVALAAQRRQAQQLGKSDELATTSMALTRVGDPVSAEYLASLDRYLDDG
GVMQVDFSRADELLNRVNGEVSRLTGGRIRDALRRSPDPLSKLLLLNAVHFKGVWAKAFSPNDTFDGVF
RGATKNTPVRMMAAKGKFRLSYDGAAYVLELPYTGDSSLVIVLPRNRLQRDLQDLEHRLQELLMAPPAEL
RSLELELPQLSLHSSLDLKDALQQMGVRALFAEHEANLSGMQPDGGLFVEDVHHEAALDVDEKGTQASA
STVAVIVSRIGTPRFSVDRPFVFALRHGPSGLLLFVGRVLDL

>s14c10_g342.t1_Chr10 XP_040067801.1 (PI=90.7) intracellular coagulation inhibitor 2-like [Ixodes scapularis]
KVLSVLFVLLAGDSECNAEDSDMKLARAQNRFALKLLKELCSKEPESDIFFSPTSISVALAMVYAAARGKS
EAEFSTALGHTAAGLPSREPTLESYKKILAEKQTNDNVSLMIANAAFVQKKLKVLKSYRKELVDIFAAMYRL
VDVGEETSARESEVDEWVKNKTKDQISGFKIPAGTITALLNAINFKGLWETPFDLKYSFSLPFYNKESEVV
MVETMSRDGTVLFTSEPSLKCEAIEHPYRGGRHSMIFVFPNENNGLAELRQAITVESIERIQRNLKETVLM
FQLPKFDLKTEYTLVPALKKLGVGSIFSDADLSGILGGEGQVVTEVQHKAAIQINEEGTVAAAATAAHAGQN
CTQSFVVNRPFLFYICEKATGRLLFLGEVRALPAIK

>s14c10_g343.t1_Chr10 XP_040067801.1 (PI=90.7) intracellular coagulation inhibitor 2-like [Ixodes scapularis]
KVLSVLFVLLAGDSECNAEDSDMKLARAQNRFALKLLKELCSKEPESDIFFSPTSISVALAMVYAAARGKS
EAEFSTALGHTAAGLPSREPTLESYKKILAEKQTNDNVSLMIANAAFVQKKLKVLKSYRKELVDIFAAMYRL
VDVGEETSARESEVDEWVKNKTKDQISGFKIPAGTITALLNAINFKGLWETPFDLKYSFSLPFYNKESEVV
MVETMSRDGTVLFTSEPSLKCEAIEHPYRGGRHSMIFVFPNENNGLAELRQAITVESIERIQRNLKETVLM
FQLPKFDLKTEYTLVPALKKLGVGSIFSDADLSGILGGEGQVVTEVQHKAAIQINEEGTVAAAATAAHAGQN
CTQSFVVNRPFLFYICEKATGRLLFLGEVRALPAIK

>s15c10_g408.t1_Chr10 XP_040067801.1 (PI=94.7) intracellular coagulation inhibitor 2-like [Ixodes scapularis]
MMLAQNVLIVLFVLLTGDGECNAEDSDMKLARAQNHFALKLLKELCSEEPESNIFFSPTSISVALAMVYAAA
RGKSEAEFSTALGHTAAGLPSRESTLESYKKILAEQQTDDNLSLMIANAVFVQKKLKVLKSYRKELVDIFAA
MYRSVDVGGGKSAMESEVNQWVKNKTKDQISGFKIPAGTITALLNAINFKGLWETPSDLKYSFSLPFYNK
GSEVVMAETMSRDGTVLFTSEPGLKCEAIEYPYKGGRHSMIFVFPNEKNGLAELRQAITVESIEKIQRNLK
ETVLMFQLPKFDLKTEYNLVPALKKLGVGSIFSDADLSGIPGGEGQVVTEVQHKAAIEINEEGTVAAAATAV
RASKNRTQSFVVNRPFLFYICEKATGRLLFLGEVHALPAIK

>s16c10_g585.t1_Chr10_Serpin38 XP_040067801.1 (PI=99.7) intracellular coagulation inhibitor 2-like [Ixodes scapularis]
MLAQKVLIVLFVLLAGDSECNAEDSDMKLARAQNRFALKLLKELCSEEPESNIFFSPTSISVALAMVYAAAR
GKSEAEISIALGHTAALLPSRESTLESYKKFLAEQQTDDNVSLMIANAVFVQKKLKVLKSYRKELVDIFAAM
YRSVDVRGEKSAMESEVNEWVKNKTKDQISGFKIPAGTITALLNAIYFKGQWETPFDLKYSFSFPFYNKGS
EVVMVETMSRDGTVLFTSEPGLKCEAIEHPYKGGRHSMIFVFPNEKNGLAELRQAITVESIEKILRNLKETV
LMFQLPKFDLKTEYNLVPALKKLGVGSIFSDADLSGIPGGEGQVVTEVQHKAAIEINEEGTVAAAATAVRAG
KNRTQSFVVNRPFLFYICEKATGRLLFLGEVHALPAIK

>s17c10_g722.t1_Chr10 XP_029835339.2 (PI=99.3) intracellular coagulation inhibitor 3-like [Ixodes scapularis]
MSPPGTFLVLIVALAAAGHVSTQSDQTKLARASSEFGLSLLKELCSTRKPQENIFFSPSSIVAALTMVYAGA
KGRSAAELETVLGLRRAKITTRDAVLGVYRTYLNDLQSPNVTVNIANAALVDKRLRILESYKGDLAKTFGAE
VRSVDFENNLKKVVSEINQWVKTKTKGKISDIVSEDSLREAVMVLMNAIYFKGSWKNAFDTKKTANFPFYN
EGIKTVQVKTMTCRSRMKYSALPELKSQAVQLPYSGDQYSMVILLPNNRTGLPQLIRALSVRTLLTLQKKL
YQYKVKLRLPKFELRTSYKLGDTLQKLGLTSIFSGKADLSGISSDVKLKVSDVVHKAMVGVSEEGTVAAAA
TIFELGALSGRRYPIPEFYVDHPFLFFICDTVKQRILFIGAVHKL

>s18ac10_g729.t1_Chr10 XP_029832440.2 (PI=99.3) intracellular coagulation inhibitor 3-like [Ixodes scapularis]
MSPPGTFLVLIVTLAAAGHVSAQSDQTKLARASSEFGLSLFKELCSTRKPQENIFFSPSSIFAALTMVYAGA
KGRSAAELETVLGLRRAKITTRDAVLGVYRTYLNDLQSPNVTVNIANAALVDKRLRILESYKRDLAKTFGAE
VRSVDFENNLKKVVSEINQWVKTKTKGKISDIVSEDSLREAVMVLMNAIYFKGSWKNAFDTKQTANFPFY
NEGIKTVQVKTMTCRSRMKYRALPELKSQAVQLPYSGDQYSMVILLPNNRTGLPQLIRALSVRTLLTLQKK
LYQDEVKLRLPKFELRTSYKLGDPLQKLGLTSIFSGKADLSGISSDVKLKVSDVVHKAMVGVSEEGTVAAA
ATKFQLGAVSGRLYPIPEFYVDHPFLFFICDTVKQRILFIGAVHKL

>s19c10_g736.t1_Chr10 XP_040070415.1 (PI=99.3) intracellular coagulation inhibitor 3-like [Ixodes scapularis]
MSPPGTFLVLIVTLAAAGHVSAQSDQTKLARASSEFGLSLFKELCSTRKPQENVFFSPSSIFAALTMVYAG
AKGRSAAELETVLGLRRAKITTRDAVLGVFRTYLNDLQSPNVTVNNANAALVDKRLRILESYKRDLAKTFG
AEVRSVDFENNLKKVVSEINQWVKTKTKGKISDIVSEDSLREAVMVLMNAIYFKGSWKNAFDTKQTANFP
FYNEGIKTVQVKTMTCRSRMKYRALPELKSQAVQLPYSGDQYSMVILLPNNRTGLPQLIRALSVRTLLTLQ
KKLYQYEVKLRLPKFELRTSYKLGDPLQKLGLTSIFSGKADLSGISSDVKLKVSDVVHKAMVGVSEEGTVA
AAATKFQLAALSGRRYPIPEFYVDHPFLFFICDTVKQRILFIGAVHKL

>s20c10_g1136.t1_Chr10 XP_002416681.4 (PI=99.7) intracellular coagulation inhibitor 1-like [Ixodes scapularis]
MSPRGTFLLSIATLAAAGHVSAQGDETELARANSEFGLSLLKELCSTRKPQENVFFSPSSIFAALTMVYAG
AKGRSAADLETVLGLRRAKITTRDAVLGTYRTYLNNLQSPSVTLKIANAALVDKRLGLLESYKRDLAETFSA
KVRSVDFQNNLKKVVSEINQWFKSKTSGKISDMASEESLRGGVMVLMNAIYFKGSWKNAFDTNQTSHFP
FYNGGIEPVQVKTMTCRSTMNYCTLPELKSQAVQLPYSGDRYSMVIVLPNDRAGLPQLIRALSVRTILTLL
KKLSPHQVKLRLPKFELSTKYKLVDALKKLGLISIFSNQADLSGISGDRNLIVSDVVHKAMVGVSEEGTVAT
AATELKFVVSSGGYPDIAEFYVDHPFLFLICDTAINQIHFIGAVHKL

>s21c10_g1137.t1_Chr10 XP_002399564.3 (PI=100) intracellular coagulation inhibitor 2-like [Ixodes scapularis]
MSPRSALIVLITVLAAAGHVSTQSDDPKLARASSEFGLTLLKELCSTRKPQENIFFSPSSIFAALTMVYAGAK
GTSAAELETVLGLSRAQITTRDAVLGAYRTYLNDLQSPNVTLKIANAALVDKRLRLLESYKRDLAETFGAEV
RSVDFENNLKNVVPEINQWVKTKTKGKISDIVSEGSLREAVMVLINAIYFKGSWENAFDTNQTAHFPFYNG
GIEPVQVKTMARLSTINYCTLPELKSQAVQLPYSGHRYSMVIVLPNDRIGLPQLIGALSVRTLLTLQKKLSP
HEVELRLPKFELRTSYKLVDALKGLGLISIFSNRSDLSGISSYGKLKVSDVVHKAMVDVSEEGTVAAAVTEV
KVVFENLSSPEALNVHVDHPFLFLISDTVQNRILFIGAVHRL

>s22c10_g50612.t1_Chr10 XP_002416681.4 (PI=99.7) intracellular coagulation inhibitor 3-like [Ixodes scapularis]
MSPRSALIVLITVLAAAGHVSTQSDDPKLARASSEFGLTLLKELCSTRKPQENIFFSPSSIFAALTMVYAGAK
GTSAAELETVLGLSRAQITTRDAVLGAYRTYLNDLQSPNVTLKIANAALVDKRLRLLESYKRDLAETFGAEV
RSVDFENNLKNVVPEINQWVKTKTKGKISDIVSEGSLREAVMVLINAIYFKGSWENAFDTNQTAHFPFYNG
GIEPVQVKTMARLSTINYCTLPELKSQAVQLPYSGHRYSMVIVLPNDRIGLPQLIGALSVRTLLTLQKKLSP
HEVELRLPKFELRTSYKLVDALKGLGLISIFSNRSDLSGISSYGKLKVSDVVHKAMVDVSEEGTVAAAVTEV
KVVFESLSSPEALNVHVDHPFLFLISDTVQNRIPFIGAVHRL

>s23c10_g1144.t1_Chr10 XP_002416681.4 (PI=100)intracellular coagulation inhibitor 1-like [Ixodes scapularis]
MSPRSALIVLITVLAAAGHVSTQSDDPKLARASSEFGLTLLKELCSTRKPQENIFFSPSSIFAALTMVYAGAK
GTSAAELETVLGLSRAQITTRDAVLGAYRTYLNDLQSPNVTLKIANAALVDKRLRLLESYKRDLAETFGAEV
RSVDFENNLKNVVPEINQWVKTKTKGKISDIVSEGSLREAVMVLINAIYFKGSWENAFDTNQTAHFPFYNG
GIEPVQVKTMARLSTINYCTLPELKSQAVQLPYSGHRYSMVIVLPNDRIGLPQLIGALSVRTLLTLQKKLSP
HEVELRLPKFELRTSYKLVDALKGLGLISIFSNRSDLSGISSYGKLKVSDVVHKAMVDVSEEGTVAAAVTEV
KVVFESLSSPEALNVHVDHPFLFLISDTVQNRILFIGAVHRL

>s24c10_g1664.t2_Chr10 XP_040074286.1 (PI=100) leukocyte elastase inhibitor-like [Ixodes scapularis]
MSPQGALMVLITALAAAGHVSTQSDDTKLARASSEFGLRLLNELCSTRKPQENIFFSPSSIFAALTMVYAG
AKGMSAAELETVLGLSRAKITTRHAVLGAYRTYLNDLQSPNVTLNIANAALVDKRLRLLESYRQDLAETFG
AEVRSVDFENNLKNVVSEINQWVKTKTKGKISDIVSEGSLREAAMVLINAIYFKGSWENAFDTNQTSNFPF
YNGGIEPVQVKTMTCRSTMNYCTLPELKSQAVQLPYSGDRYSMVIVLPNDRTGLPQLIGALSVRTLLSLLK
KLSPHEVELRLPKFELRTSYKLVDALKRLGLISIFSNRSDLSGISSQGKLKVSDVVHKAMVDVSEEGTVAAA
ATEVKVVFENLSYPDALDFHVDHPFLFLISDTVQNRILFIGAVHRL

>s25c10_g1669.t1_Chr10_Serpin15-like XP_029835338.2 (PI=99.5) intracellular coagulation inhibitor 2-like [Ixodes scapularis]
MSPRGTFLLLIATLAAAGHVSAQGDETELARANSEFGLSLLKELCSTRKPQENVFFSPSSIFAALTMVYTG
AKGRSAADLETVLGLRRAKITTRDAVLGTYRTYLNDLQSPNVTLKIANAALVDKRLRLLESYKRDLAETFGA
EVRSVDFQNNLKKVVSEINQWFKSKTSGKISDMASEESLRGGVMVLMNAIYFKGSWKNAFDTNQTSHFP
FYNGGIEPVQVKTMTCRSTMNYCTLPELKAQAVQLPYSGDRYSMVIVLPNDRAGLPQLIRALSVRTILTLL
KKLSPHQVKLRLPKFELSTKYKLVDALKKLGLISIFSNQADLSGISGDRNLIVSDVVHKAMVGVSEEGTVAT
AATEVKFLVSSGGYPDIAEFYVDHPFLFVICDTATNQIHFIGAVHKL

>s26c10_g1716.t1_Chr10 XP_029832440.2 (PI=99.8) intracellular coagulation inhibitor 3-like[Ixodes scapularis]
MSPPGTFLVLIVTLAAAGHVSAQSDQTKLARASSEFGLSLFKELCSTRKPQENIFFSPSSIFAALTMVYAGA
KGRSAAELETVLGLRRAKITTRDAVLGVYRTYLNDLQSPNVTVNIANAALVDKRLRILESYKRDLAKTFGAE
VRSVDFENNLKKVVSEINQWVKTKTKGKISDIVSEDSLREAVMVLMNAIYFKGSWKNAFDTKQTANFPFY
NEGIKTVQVKTMTCRSHMKYRALPELKSQAVQLPYSGDQYSMVILLPNNRTGLPQLIRALSVRTLLTLQKK
LYQDEVKLRLPKFELRTSYKLGDPLQKLGLTSIFSGKADLSGISSDVKLKVSDVVHKAMVGVSEEGTVAAA
ATKFQLGAVSGRLYPIPEFYIDHPFLFFICDTVKQRILFIGAVHKL

>s27c10_g1726.t2_Chr10 XP_029841671.2 (PI=99.5) intracellular coagulation inhibitor 3-like[Ixodes scapularis]
MLAEKVFLVLFVLLAGDSECYAEDSDMKLARAQNHFALQLLKELSSEAPESNIFFSPTSISVALAMVYAGAR
GKSEAELSSALGHTAAGLSNRESILESYKKILAKQQTDDNVSLMIANAVFVEKTLKVLESYQKELVDTFAAM
FRSVDVGAEKSAMESEVNEWVKNKTRGKISGFQIPADTVMALLNAIYFKGLWNTSFDPNNTSPLPFHNKG
SEVVMVETMTRYGKVPFTSAPDFEAIELSYKGDRHCMVIVLPHEKKGLPKLRDAITVETIKKIQESLKNETV
KIQLPKFNLETEYGLIPALRKLGVRSIFSDADLSGITGGRGLRVTEVQHKAAIEVNEDGTVAAAATVVLINKS
GPPSFAVNRPFLFYIREKATGRVLFLGEVHELPAAKPTLG

>s28c10_g3088.t1_Chr10 XP_040063232.1 (PI=100) intracellular coagulation inhibitor 3-like[Ixodes scapularis]
MLAEKVFLVLFVLLAGDSECYAEDSDMKLAQAQNHFALKLLKQLSSEAPESNIFFSPTSISVALAMVYAGA
RGKSEAELSSALGHTAAGLSSRESILESYKKILAKQQSDDNVSLMIANAVFVEKNLKVLESYQKEVADTFG
AMLRSVDGGAENSAMESEVNEWVKNKTRGKILGFEIPPDVVIVILNAIYFKGLWETPFKSNNTSPLPFYNK
GSQEIKVETMTRDGKVPFTYEPDFAAIELSYKGDRHCMVIVLPHEKKGLPKLRDAITVESIKKIQGSLTNET
VEIQLPKFNLETDYRLIPALTKLGVRSIFSDADLSGMTVDRGLRVTEVQHKAAIEVNEEGTVAAGATEVEGG
RGGPRPFTVNRPFLFYIREKATDRLLFLGEVHELPAVKPALG

>s29c10_g3305.t2_Chr10_Serpin29 XP_040063232.1 (PI=100) intracellular coagulation inhibitor 3-like[Ixodes scapularis]
MMLAEKVFFVVFVLLGGNSIGNSEDSDMKLAQAQNHFALKLLKELCTKHQESNIFFSPTSIFVALAMVYAG
ARGKSEMELSAALGHTAAGLSTRKSILDSYKKILTEQQTDDNVSLMIANAVFVQKNLKVLESYQKELADTF
AAMFRSVDVGAENFAMESEINEWVKNKTRGKISGFKIPSDTVVALLNAIYFKGIWKTPFEPKYTSPLPFYN
KGSEEVKVETMTRTSMVPITFEPDFEAIELSYKGDGHCMVIILPRNHTSKALAELRDSMTLESIEKIKESLK
RESVTIQLPKFHLKTEYGLIPALKKLGVRSIFSDADLSGITGDGGLRVTDVQHKAAIEVNEEGTVAGAATDV
VLGRILPTRFFVNRPFLFYIREKATGRVLFLGEVHELPAAKVKPALRQCDSDPTYGDEIVFQI

>s30c10_g3308.t1_Chr10 XP_029832862.2 (PI=99.8) intracellular coagulation inhibitor 3-like [Ixodes scapularis]
MLAGKVFLILFVLLAGDSECCTEDSDMKLVRAQNHFALKLLKELSSEAPESNLFFSPTSISVALAMVYAGAR
GKSEAELSTALGHTAAGLSSRESILASYKTILAQQQTDDNVSVMIANAVFVQKNLKVLESYQKELVDTFAA
MFRSVDVGTEKSVMESDVNQWVKNKTRGKISGFKIPANTIMALLNAIYFKGLWETPFHLNNTVPLNFYNK
GSKKIKVEFMQRCGNVPISFGPNYAAIELSYKGDKHGMVIVFPYKIIGLPKLQDAMTLETIERIRGSLKSEAV
WVRLPKFDLKTEYGLIPALKKLGVRSIFSHADLSGITGDRGLWVTEVQHKAAIEVNEEGMGVSAPMKLEN
EGRFPTPFPVNSPFLFYIHEKATGRVLFLGAVHELPVAKVKPALR

>s31c10_g3314.t2_Chr10_Serpin41-like XP_029841035.2 (PI=97.3) intracellular coagulation inhibitor 3-like [Ixodes scapularis]
MLAEKLFLGLFVLLAGDSECYAEDSDMKLARAQNHFALKLLKELCSENPETNIFFSPTSISVALAMVYAGAR
GKSEAELSSALGHTAAGLSSRESILESYKKILAKQQTDDNVSLMIANAVFVEKTLKVLESYQKELVDTFAAM
FRSVDVGAEKSDMESEVNEWVKNKTRGKISGFEIPADTVMALLNAIYFKGLWKTSFEPEYTSLLPFFNKG
SQEVKVETMTRRGKVPVTYEPDFAAIELSYEGDGHCMVIILSRNLTPNGLSELRDAMTIESIEKIQESLKEE
SVRIQLPKFDLKTEYGLIPALEKLGVRSIFSDADLSGITADGGLRVTDVQHKAAIEVNEEGTVAGAATEVKE
GRILISFVVDRPFLFYIREKATGRVLFLGEVHELPAAKVVPAPC

>s32c10_g3318.t1_Chr10 XP_042144458.1 (PI=83.6) intracellular coagulation inhibitor 3-like [Ixodes scapularis]
MLAEKVFPVLFILLPGDSECYAEESDMKLARAQNHFALKLFKELGSKDPESNLFFSPTSISVALAMVYAGA
RGKSEAELSTALGHTAAGLSSRESIMDSYKNILAKQQTDDNVSLMIANAVFVKKTLKVLESYRKELVDIFAA
MLRLVDVGAVNSGMESEVNEWVKNKTRGKILGFKIPADTVMALLNAIYFKGLWKTPFQPNNTSPLPFYNK
GSEEVKVETMTRVGMVPFTSEPDFAAIELSYEGDGHCMVIILPRQRKGLPKLRDAITVESIEKIQGSLKEET
VKCQLPKFDLKTEYGLVPALKKLGVRSIFSDADLSGISGDRGLTVTDVQHKAAIEVNDEGTVAAAATVIIFRR
YPTPFVVDRPFLFYIREKATGRLLFLGEVHELPAAKPSVS

>s33c10_g3321.t1_Chr10 XP_042144458.1 (PI=85.4) intracellular coagulation inhibitor 3-like [Ixodes scapularis]
MLAEKVFPVLFLLLAGDSECYAEESDMKLARAQNHFALKLFKELCSKDPESNLFFSPTSISVALAMVYAGA
RGKSEAELSTALGHTAAGLSSRESIMDSYKNILAKQQTDDNVSLMIANAVFVKKTLKVLESYRKELVDIFAA
MLRLVDVGADNSDMESEVNEWVKNKTRGKISGFKIPADTVMALLNAIYFKGLWKTPFKPNNTSPLPFYNK
GSEEVKVETMTRVGMVPFTSEPDFAAIELSYKGDGHCMVIILPRERKGLPKLRDAITVETIEKIQGNLKEET
VKIQLPKFDLKTEYGLVPALKKLGVRSIFSDADLSGITGDRGLTVTDVQHKAAIEVNEEGTVAAAATVIIFRR
YPTPFVVDRPFLFYIREKATGRLLFLGEVHELPAAKPTVG

>s34c10_g3396.t1_Chr10 XP_040075471.1 (PI=90.2) intracellular coagulation inhibitor 3-like [Ixodes scapularis]
MLAKKVFLVLFVLLAGDSECCAEDSDIKLAQAQNHFALKLLKELSSEAPESNIIFSPTSISVALAMVYAGARG
KSEAELSSALGHTSAGLSSRESILESYKKILAKQQTDDNVSLMIANAVFVKKTLKVLESYQKELIDTFEAMF
RSVDGGAEKYDMQSEVNEWVKNKTRGKISGFEIPAGVVMVILNAIYFKGLWETPFKPNNTSPLPFYNKGS
QEIKVETMTRLGKVPFTSAPNFEAIELSYKGDRHCMVIVLPHEKKGLPKLRDAITVETIKKIQGSLKNKTVKI
QLPKFNLETEYGLIPALTKLGVRSIFSDADLSGITGDRGLRVTDVRHKATIEVNEEGTVATGVTKVDIGRSLP
RRFIVDRPFLFYIREKETGRVLFLGEVHELPAAKPVLG

>s35c10_g3397.t1_Chr10 XP_040075471.1 (PI= 90.5) intracellular coagulation inhibitor 3-like [Ixodes scapularis]
MLAKKVFLVLFVLLAGDSECYAEDSDIKLAQAQNQFALKLLKELSSEAPKSNIFFSPTSISVALAMVYAGAR
GKSEAELSSALGHTTAGLSSRESILESYKKILAQQQTDDNVSLMIANAVFVQKNLKVLESYQKELVDIFAAM
FRLVDGGAEKSDMQSEVNEWVKNKTRGKISGFETPADVVMVILNAIYFKGLWETPFKPNNTSPLPFYNKG
SQEIKVETMTRLGKVPFTSAPDFEAIELSYKGDRHCMVIVLPHEKKGLPKLRDAITVETIKKIQGSLKNETVK
IQLPKFNLETEYGLIPALRKLGVRSIFSDADLSGITGDRGLRVTDVRHKATIEVNEEGTVATGVTKVDIGRSL
PRRFIVDRPFLFYIREKETGRVLFLGEVHELPAAKPVLG

>s36c10_g3610.t1_Chr10_Serpin24-like XP_029839156.2 (PI=100) intracellular coagulation inhibitor 3-
like [Ixodes scapularis]
MKLAREQNHFALKLLKELSSEQPESNLFFSPTSISVALAMVYAGARGKSEAELSTALGHTAAGLSSRESILA
SYKKILAEQQIDENVSLMIANAVFLKKTLKVLESYQKELVDIFAAMFRSVDVGAENSAMESEVNEWVKNKT
KGKISGFKIPANTIMTLLNAIYFKGLWNTSFDPNYTSPLPFYNKGSEEVKVETMTRSGWVPFTSEPDFAAIE
LSYKGDRHSMVIVLPSEKRGLAKLRQAMTVESVRKIHVSLKQRFVRIQLPKFDLKTEYSLVPALKKLGVASI
FSDADLSGITGNGGLVVSGVLHKAAVEVNEEGTVATGVTVVATAMSRPPSFAVNRPFLFYICEKATGRVLF
LGEVHELPAAKVKPTLR

>s37c10_g3676.t1_Chr10 XP_040066725.1 (PI=98.7) leukocyte elastase inhibitor [Ixodes scapularis]
MKASTVWFLLAAVVVCVVQGDEVQLARANNEFALNLLKELSSRKPGGNVIFSPTGIYEALAMVYAGSRGE
SQAELSKVLGHDGAGLKNRDAVLSAYKKLYAFKNDSEMNVTLNVVNAVLVQEELRVLDGYKTELAEVFGA
KLKTVDFFNESSRVSSEISQWVRQKTGGKIKSVLPDGIPMNTVMLLLNAVYFKGTWLIKFDPEQTVQRPFY
NHGTKFVAKDTMVLRGEIKHAWLLELDAQAVELPYKDERFAMVIVLPNSKTGLPKLRDSFSLKLMDEIDEE
LDVEKVHLKLPKFELKAGYDLVPSLQRLGLKSVFTSDADLSGISGSRDLAVSDVKHAAVVEVNEEGTAAAS
ATSAGIVAKSIPQTANFYVDHPFLFYVHDVESKRVLFMGEVHEL

>s38c10_g3680.t1_Chr10_Serpin13 EEC20552.1 (PI=99.8) serpin-2, putative [Ixodes scapularis]
MSVAKAVLVLTVLVSSYSASDDVALARATNVFGINLLRELSSVKPRQNIVFSPSSISVEVAMVYAGAAGQCA
EETSRVLGHIGVGLLDRDTVLASYKKRINDTSDILMMANAVLVRNTSSVLDTYKRTLQEVFAAQFKSVDFL
AYGYRMAAEINEWMCEKTEGRVTKGILDGMPMNTVVYVVSAASLATLPWISQFEPNNTRILPFFNHGIELA
RREVMTATANIRHAKLPDLESEAIELLGTDLRRSMVIVLPDSITGLSKLRATFNAQNIDDIDTELRSQRVNLR
LPKFKINNRHNLVPTLRGLGINCVFGTEPDLSKMSCDKNLYVSEIAHVAVMTVNEEGAGFINPTPGWTPNA
ERAMRLRPLDFYVDHPFLFYIRDRLANTILLMGEVHEL

>s39c10_g3676.t3_Chr10_Serpin14-like XP_040066725.1 (PI=97.5) leukocyte elastase inhibitor [Ixodes
scapularis]
MKASTVGFLLAAVVVCVVQGDEVQLARANNKFALNLLKELSSRKPGGNVIFSPTGIYQALAMVYAGSRGE
SQAELSKVLGHDGAGLKNRDTVLSAYKKLYAFKNDSEMNVTLNVVNAVLVQEELRVLDRYKTELAEVFGA
KLKTVDFFNESSRASSEINQWVRQKTRGKIQSVLPDGIPMNTVMLLLNAVYFKGTWLIKFDPEQTVQRPF
YNHGTKFVAKDTMVLRGEIKHAWLLELDAQAVELPYKDERFAMVIVLPNSKTGLPKLRDSFSLKLMDEIDE
ELDVEIVHLKLPKFELKAGYDLVPSLQRLGLKSVFTSDADLSGISGSRDLAVSDVKHAAVVEVNEEGTAAA
SATSVGIVAKSIPQTANFYVDHPFLFYVHDVESRRVLFMGEVHEL

>s40c10_g3685.t1_Chr10_Serpin36-like XP_029826743.3 (PI=97.9) intracellular coagulation inhibitor 2-
like [Ixodes scapularis]
MNMKNLVFLLSVLVISWAQMDDRLTLANNRFGLRLFGVLSSTPDSNVFFSPYSVSTAMAMAYAGVRGEA
QQELSQRLEFSAAGLSEEQVLDAYAQHTRRILLVQTNSTLKVANAAAVHGGLALLASYERTLSRSFYSELL
NVDFANEADAAVSFINGWVRQRTQNKIDGIIDGKLDPNTRLMLLNAIYFKGAWNRQFNASLTAKGPFFNG
GTAPVQVDVMTGTFDIPYGYFEDLQVDMAELPYRGLDFSMSILLPRHSNALEALKRNLTAELFQSMVSRL
RERKVNVALPKFKLDTKYLLKEALQTLGIRKIFTSGSDWSGITTDNLSAVSTVIHKAAVEVNEEGSVAATT
GIGIVAISLPPPPVEFRVNHPFLFFIRNTNTNDILFVGHINAL

>s41c10_g3686.t1_Chr10_Serpin39 XP_002402368.4 (PI=98.5) intracellular coagulation inhibitor 1 [Ixodes scapularis]
MKTLAAFLSLLVLCWAQEEAKLTKANNRFGLRLLRTLPSGPEENVFFSPYSVSTAMGMAFAGARGQTQQ
ELSQGLGFSDVDLTDAGVLDAYTRHTERLKSTASNSTLDVANAAAIQRTLALLNSYESALQSSFGAELHKV
DFAGESQAAVDFVNNWVKRKTYDKIEKLFNEPLDSDTLLVLLNAIYFKGDWGTAFNKEHTEKRQFFNGGV
TPVEVDTMRLEARIKYRFFDDLQVEVVELPYRGLDYTMAILLPKENTGVEGLKQNLTIDRFQNYLSDLRER
KITVLLPKFKLETKYSLKTPLQSLGIKQIFEPGADLSGINDESLRVSAVEHKAVVEVNEEGTEAAAATGVVIV
PYSLGPEPVVFRVDHPFLFFIRNTRTDDIFFMGQVNKL

>s42c10_g3687.t1_Chr10_Serpin34-like XP_040356992.2 (PI=98.2) intracellular coagulation inhibitor 1 [Ixodes scapularis]
MMLGRLAALLVLVVSCKAQEELKLTLANNRFGLRLFHALPSAAEVNVFFSPYSVSTAMGMTYAGARRDTA
EELFRGLGYSASGLTAPQVLDSYARHTQRLLSSESQSTLKVANGAAIQENLTLLDSFRNTLESSFNAEVHQ
VDFVHRQQDAFEFINRWVKQKTSDKIDKLFDEPLSPATKLVLLNAIYFKGVWNTQFDRALTRRRQFWNGG
VSPILVDTMMQNMHVGYGSFGDLYVDVAELPYRGRDYSMVILLPKRNDGVDRLKGNLTVEILRSLLSELR
ERDVDVFLPKFKLESTYQLEDFLTALGIRQIFSNGADLSGVSDRNLRVSAVVHKAVLEVSEEGSEASSATG
VVILPGSVMPVAFVADHPFLLFIRNTRTNDIIFAGQVNNL

>s43c10_g3689.t1_Chr10_Serpin40-like XP_040066693.3 (PI=97.9) intracellular coagulation inhibitor 1-like [Ixodes scapularis]
MRVIFVILPFCAVVLGEKDKLTTANNHFGIRLLQNLPSSGEENLFFSPYSVSMAMGMAYAGAKGKTQEELL
RGLGYSQSGLTRDEVLDAHVRHSQNFLPGASNTTINVFNGAAVDESLSVLPAYKESIKNVFQAELHKVDF
KQKGQEAVDIINEWVKNRTEQKIKKLFSEPPRPDTRMLLLNAIYFKGVWSTGFNEKLTRKRRFFNGGLTPT
EVDTMQGVFNTHHNFFDHLQLDMVDLPYSGHDYSMTIMLPQKKDGVEALKGNLTVNLFEHLFAELRERE
VYLTLPKFKFETEYSLKEPLNNLGIRNIFSDVADLSGISDHEALRVSKVVHKAVIEVNEKYSEAAAVTAVAVP
RIVGGDLTAHVSVDHPFLFFIRNKDTSDILFAGQVNKL

>s44c10_g3691.t1_Chr10_Serpin34-like XP_040356992.2 (PI=94.1) intracellular coagulation inhibitor 1 [Ixodes scapularis]
MMLGRLAALLVLVVSCKAQEELKLTLANNRFGLRLFHALPSAAEVNVFFSPYSVSTGMGMTYAGARRDTA
EELFQGLGYSASGLTAPQVLDSYARHTQRLLSSESQSTLKVANGVAIQENLTLLDSFRNTLESSFNAEVYQ
VDFVHRQQDAFEFLNRWVKRKTSDKIDKLFDEPLSPSTKLVLLNAIYFKGVWNTQFHRDFTQKRQFWNG
GVSPTLVDTMMQNMYVGYGSFGDLDVDVAELPYRGRDYSMVILLPKRNDGVDRLKGNLTVEIMRSLLSE
LRERDVVVFLPKFKLESTYQLEGPLAALGIRQIFSNGADLSGISDRNLRVSAVVHKAVLEVSEEGSEASSAT
GVLILPGSGMPVEFVADHPFLLFIRNTRTNDIIFAGQVNKL

>s45c10|JAA_g51539.t2_Chr10|JAA_Serpin3 XP_029826745.3 (PI=98.48) intracellular coagulation inhibitor 3 [Ixodes scapularis]
MKYLVTFLAFLALSWAGDEDKVTLANNRFCFELLQSLQGSIDTNIFFSPYSVSTAMGMIYVGARGETQREL
FQGLGYSVPGLTHDQLLSAYGPHTSRLQSLQSNTTFKVANAVAIDERLAIEESYENTLTSSFKADLHKVEFL
HGPQATLNLINDWVKEKTDGKIMGLFDGQLDRSTRLVLVNAIYFKGSWNRPFDRILTEKREFFNGGETRTE
VDTMVGRFEVGYHVSDQLRVAVADLPYVGHDFSMTVLLPLENDGVERLRRNLTLDLFQSLFSELRGREV
DVFLPKFKLDTKNILNEPLKRLGIRKIFGGGTDLSGINGDTDLVVDAVVQKAVVEVNEEGSEAAGATGAGL
MPLSAAVSPPPVFRVDHPFLFFIRNTRTRDILFAGQVNKL

>s46c10_g3697.t1_Chr10_Serpin1 XP_042145815.1 (PI=98.9) intracellular coagulation inhibitor 1 [Ixodes scapularis]
MRHLIIFVSLLVACWSQKEEQLTVANNRFGLQLLKILQSSPQGNVFFSPYSVSSVMGMAFAGARGDTQHE
LSQGLGYASAGLQDSDILDAYALHTHRLRSLESNSTLEVANAAAVHERFALQSAYELHLAGSLTAMLLKVD
FENGGRDAVDTINRWVKLRTHEKIPRLFNAPLESSTRLVLLSAIYFKGKWEKEFEKNHTEKRTFLNGGTTP
TQVDTMTGLIPVRHQSFESLGVDMAELPYQGGDYSMVILLPKQNDGVEVFKQNLTDVLIKDLASQLVDRQ
VRVFLPKFKLEAEYSLRNPLQNLGIRRIFGPGADLSGITEDNNIQVSAVVHKAFVKVNEEGTEAAAVSGAVF
VTKTLGLPTVEFKVDHPFLFFIRNTRTKDVMFAGQVNNL

>s47c10_g3698.t1_Chr10_Serpin2-like EEC19558.1 (PI=93.1), serpin-4 precursor, putative [Ixodes scapularis]
MKTLVVFMCLMVAGWARYENEMRLANNRFAVDLIKSLPSSPEKNIFFSPYSISTAMGMVFAGARGKTLKN
LYDGFGYLRSGLKEDWVLQAYADHAKQLQVGQSQSTFDVANAAAIHERMSLLNAFESTLDSTFHAQLLKV
DFVNGGPAAVDEINRWVKQKTHDKIDELFDDPLDPDTRLVLLNAIFFKGVWSTKFDESATTEKQFLNGGTT
PTQVDTMTKSIHIGYKSFPTMRLEVAELPYAGGNYSMVILLPRGNEGIEGFKHNLTENHLQDIIGHVEPRKV
TVSLPKFKLEAEYSLKDNLKNLGITEMFSAQADLSGITSDADLTVSDVVHKAVVEVNEEGTEAAAASGVVA
VNRLIGVPSLEFNVNQPFLFFIRNTQTQDLLFAGQVNHL

>s48c10|JAB_g3698.t2_Chr10|JAB_Serpin3 XP_029826744.3 (PI=99.5) intracellular coagulation inhibitor 3 [Ixodes scapularis]
MKYLVTFLPLLVLSSAGDEDKVTLANNRFCFELLQSLEGFTETNILFSPYSVSTAMGMIYVGARGETQREL
FQGLGYSASGLTHEQVLSAYGPHTSRLQSPQSNATFKVANAVAIDERLALEESYENTLTTSFKADLHKVEF
LHGPQATLNLINDWVKEKTDGMIMRLFDGQLDRSLRLMLLNAIYFKGSWNRPFDKTLTEKRQFFKFGGGL
RTEVDTMVGRFEVGYHMSDQLRVAVADLPYVGHDFSMTVLLPLEKYGVEQLRRNLTLDLFQSLVSELRG
REVDVFLPKFKLDTKYVINEPLIRLGIRKIFGGGTDLSGINGDTDLVVNVVLQKTVVEVNEEGSEAAGATGA
GLMPLSAAVSPPPVFRVDHPFLFFIRNTRTKDILFAGQVNKL

>s49c10_g3699.t1_Chr10_Serpin4 XP_040066711.2 (PI=96.7) intracellular coagulation inhibitor 2 [Ixodes scapularis]
MKTLIVLMCSLVVVWARYENEMRLANNRFAVDLLRGLPSSPEKNIFFSPYSISTAMGMVFAGAEGETLKDL
YDGFGYLRSGLKEDWVLQAYADHAKQLQVGQSQSTFDVANAAAIHERLALLSTYENTLDSTFHAQLLKVD
FVNGGPAAVDEINRWVKQKTHGKIDKLFDGALDPLTRLVLLNAIFFKGAWSTKFDESATTKKQFLNGGTTP
TQVDTMTNRIRIGYKSFPALRLEVVELPYAGGNYSMVILLPRGSEGIEGFKHNLTEHLLQDFIGHVEPREVT
VSLPKFKLETEYSLKDNLKRLGITLIFGAQADLSGIAGGGDLVVSDVVHKAVVEVNEEGTEAAGVSGVVVN
TRLVEVPFLEFNVDQPFLFFILNTHTKDLLFAGQVNHL

>s50c10_g3700.t1_Chr10_Serpin5-like ABI94057.1 (PI=95.2) serpin-4 precursor [Ixodes ricinus]
MRSFATFMSLLTICWGLHEDRLTLANNRFAISLLHGLPTSTETNIFFSPYSISVALGMAFAGARGETREDLF
QGFGYARSDIEDDAVLEAYASHTRRLRSLRSNSTLDAAIGAAIHERISLLSSYENVLNNSFGADLLKVDFIN
GGQAAVDVINGWVHGKTRGKINLLFGEPLETITQLVLLNAVYFKGTWDMVFDQRLTTKKPFMNACSTPTE
VDTMRGEVYVRHKSFPLLGVDIAEIPYRGMDYSMTILLPTRIDGAEALKRNITEHLLQDLLKQLVEQQVTVY
LPKFKLETEYLLKDHLKKLGINRIFGSGADFSGITHDANLVVSDVVHKTVLEVHEAGTEAAGATGVIIVAESL
VESVEFRVDHPFIFFIRNTQTKDILFVGQVNHL

>s51c10_g3701.t1_Chr10_Serpin6 AID54718.1 (PI=99.2) blood meal-induced serine protease inhibitor [Ixodes scapularis]
MKVLVTFLSVLALCWAGEEDKLTVASNNFGLRMFPLLPSSPETNIFFSPCSLLIAMGMAYAGARGETQKELYENLGYSNAGLAEGQVLDAYTRQTQKYQSLQSNTTVDVANAAAIHLRLSLLDGYENALRNSFNAQLQKVDFVDGGQAAIDTINKWVKEKTHNKIEALFSEPLDPLTRFVLLNAMYFKGTWNTEFDEKRTEKRPFLNGGVTPAEVDTMVGKIRIRHNSFENLGVDVAELPYRGGDYSMVILLPREKTGVEALKRNLTADLLDTLVHQLVEREVDVFLPRFKLETMYSLKEILQQMGIKKIFDGADLSGITGDKSLEVSAVVQKAVVEVNEEGTEAAVVSGVIGATRVASFPSFNFIVDHPFLFFIRNTHANAILFAGQVNHL

>s52c10_g3702.t1_Chr10_Serpin7 XP_029826754.3 (PI=97.7) intracellular coagulation inhibitor 2 [Ixodes scapularis]
MKVITAFLSVFVLCSAEDDDKLTVASNDLGMRLLPLLPSSPEENIFFSPYSLSIAMGMAYAGAGGETRQELQENLGYSRAGLPEEQVLDAYARQTQRHLSDPSNTTVDVANTAAIHLGLPLLNEYEATLRNSFNADLQKVDFVENGQGAVDVINSWVKDKTHNKIESLFSEPLDPLTRFVLLNAMYFKGTWKTEFQKRRTGQRSFFNGGVTQAQVDTMIGKIRIRHNSFNDMSVDVAELPYRGGDYSMVILLPQEKTGVEALKMNLTAEILKTILDRLVQRDVTVFLPKFKFENKYSLKEILQNMGIRRIFGGDADLSGISGDTSLQVFDVVQKAVVEVNEEGTEAAVVSAVIGGLRSGSFGGFEFKVDRPFLFFIRDTRSNAILFVGQVNHL

>s53c10_g3703.t1_Chr10_Serpin8-like EEC19554.1 (PI=99.4) secreted salivary gland peptide, putative [Ixodes scapularis]
MKVLTLFLSTFALCWAEEDDKITSASNNLGLRLFPLLPSPPEENVFFSPCSLSVAMGMAYAGARGETRQELYDHLGYSSAGLPEAQVLDAYARQMQRHQPGQSNTTIDVANSAAIHLTLSLLNEYENTLKNSFKADLQTVDFVDGGQAAVDVINRWVKEKTHDKIESLFSAPLSPLTRFVLLNAIYFKGTWETEFEKKNTRNMPFLNGGVTQAEVQTMVEKIRIRHNSFEDLGVDVAELPYRGGDYSMLILLPKEKTGVEVLKGNLTAGLLQTLIDRLVQREVTVFLPKFKLETKYYLKEFLQKLGIKRIFGKDADLSGISEDASLKVSAVVQKAVVEVNEEGTEAAVVSGVVGAIRASAPQRSFEFRVDHPFLFFIRNTRTNGLLFMGQVNML

>s54c10_g3706.t1_Chr10_Serpin9 XP_002415886.4 (PI=99.2) intracellular coagulation inhibitor 1 [Ixodes scapularis]
MRAVMKTLVPLLFFLVSCRAQGNDKLTLANNQFGLRLLNTLPSPPEENVFFSPYSISTALGMAYAGARGDTQEELSQQLGYTAAGLSQDDVFNAYSDHTQWLKASRSNSTLSVANAAVLHDKVGLRYTFQRTIDHAFDADILKVDFVNERKGAMDRINYWVKDKTNGKIRSLFNKPLESETRLLLLNAIYFKGSWNTKFNKSRTEKREFLNGGVTPTKVDTMMGSINIGHRFFTDLQIDVADFPYQGQDYSMTVILPWRNDGVEAIKQNLTLDLFQKLVSELRERRVFVYLPKFKIEAEYSLKEPLQQLGIKQIFSGGSDLSGVTNDNDLVVSAVVHKAILEVNEEGSEAAAVSSVVAVTRIGTQAFEFNVDHPFLFFIRNTVTNDILFAGQVNSF

>s55c10_g51546.t1_Chr10_Serpin10 XP_042145816.1 (PI= 95.6) intracellular coagulation inhibitor 3-like [Ixodes scapularis]
MIIALILPVAFAVAHGQMSDDERLVNANNEFGLNLLRELSSTRPGCNVFFSPSSVSAALAMVYAGAGGLTEAELSATLGHAMMDLTDRTALMSAYKRLLADNQYQDVALDIANAVLIRKNFRVLDGYKRDLADVFKANLTSVDFAHEGSKVASEIDQWVKQKTKGKIQNIVGGGLPEDTAMFLISAVYFKGTWVTKFDSAKTKPRPFYNHGTESSDRQTMELMSQVKFGRLDSLRSRAVEIPYKGDRVSMVVLLPDSNTGLPRLRDGLTAEVLKELEDRMWWTTVELRLPKFEQAKEYNFVPILQKLGLKSVFTANADLSRAADPGLFVSDVKHKAMIEVSEEGTVAAAVTTVRNVNGGRGRSIAVPQIRRFHVDHPFLFYIRDKVTNRVLFMGEINRL

>s56c10_g3708.t1_Chr10 EEC19552.1 (PI= 93.0) serpin-2, putative [Ixodes scapularis]
MIIALILPVAFAVAHGQMLSDDERLVNANNEFGLNLLRELSSTRPGCNVFFSPSSVSAALAMVYAGAGGLT
EAELSATLGHAMMDLTDRTALMSAYKRLLADNQYQDVALDIANAVLIRKNFRVLDGYKRDLADVFKANLTS
VDFAHEGSKVASEIDQWVKQKTKGKIQNIVGGGLPEDTAMFLISAVYFKGTWVTKFDSAKTKPRPFYNHG
TESSDRQTMELMSQVKFGRLDSLRSRAVEIPYKGDRVSMVVLLPDSNTGLPRLRDGLTAEVLKELEDRM
WWTTVELRLPKFEQAKEYNFVPILQKLGLKSVFTANADLSRAADPGLFVSDVKHKAMIEVSEEGTVAAAVT
TVRNVNGGRGRSIAVPQIRRFHVDHPFLFYIRDKVTNRVLFMGEINRL

>s57c10_g3709.t1_Chr10_Serpin11-like XP_029826755.3 (PI= 98.2) intracellular coagulation inhibitor 3-like [Ixodes scapularis]
MLSLNTFQRLGVILALIIDCSCECPKAKFTNATTDFGFKLLRSSFANTNENMNVVLSPTGVATLLAIVYAGAG
GKTAEELSTLLSYTDSELNRIEAVLACQRNILDDPQNNSTAFDVADVILVRSNVTILQNYRKTVLTDFATEIVG
VNFEKAGAKVVSDTNDLVAERTSGKITNIIGGELGADDQMVLLNAAYFNGSLKMPSSTSYSAQLPFYTEGI
HLTSAETIVCTGPMLYTSETGLNSQVVELPHTDGRHSLGIVLPHTKRGLSNLTHAMDARSFAEMRKNLKPV
TVSLTLPRFNFTTTCDLAGPLIRLGAASLFSDADLTGITSDKGLRVTEMRHKARIEVTEERAVSPGGGAIAA
PKAVPVFFHARHPFLFYVRDHRTERTILVGLVQQL

>s58c10_g3713.t1_Chr10 XP_002415886.4 (PI=97.9) intracellular coagulation inhibitor 1 [Ixodes scapularis]
MRAVMKPLVPLLFLLVSCRAQGNDKLTLANNQFGLRLLNTLPNSPEENVFFSPYSISTALGMAYAGARGDT
QEELSQQLGYTAAGLSQDDVFNAYSDHTQWLKASRSNSTLSVANAAVLHDKVGLRYTFQRTIDHAFDADI
LKVDFVNERKGALDRINYWVKDKTNGKIKSLFNKPLESETRLLLLNAIYFKGSWNTKFNKSRTEKREFLNG
GVTPTKVDMMMGSINIGHRFFTDLQIDVADFPYQGRDYSMTVILPWRNDGVEAIKQNLTLDLFQKLVSELR
ERRVFVYLPKFKIEAEYSLKEPLQQLGIKQIFSGGSDLSGVTNDNDLVVSAVVHKAILEVNEEGSEAAAVSS
IVAVTRIGTQAFEFNVDHPFLFFIRNTVTNDILFAGQVNSF

>s59c10_g52506.t2_Chr10 XP_040067905.1 (PI= 97.7) intracellular coagulation inhibitor 3-like [Ixodes scapularis]
MLAKKVSLILFVLLARDNECYAEDSDMKLAQAQNHFALKLLKELSSEAPESNIFFSPTSISVALAMVYAGAR
GKSEAELSSALGHTATGLSSRKSILEFYKKILAQQQTDDNVSLMIANAVFVEKNLKVLESYQKELVDTFAAM
FRSVDGGAEKSDMQSEVNEWVKNKTRGKISGFETPADVVMVILNAIYFKGLWETPFKPKNTSPLPFYNKG
SQEIKVETMTRIDNVPFTFEPDFAALELSYKGDRHCMVIVLPREKKGLPKLRDAITVETFKKIQGSLKNDTV
QIQLPKFNLETDYSLVPALTKLGVRSIFSDADLSGITGDRGLRVTEVRHKAAIEVNEEGTVATGVTKVDMGR
RIAGHFIVDRPFLFYIREKETGRVLFLGQVHELPAAKLVLG

>s60c10_g52507.t1_Chr10 XP_040067905.1 (PI= 97.5) intracellular coagulation inhibitor 3-like [Ixodes scapularis]
MLAKKVSLILFVLLARDNECYAEDSDMKLAQAQNHFALKLLKELSSEAPESNIFFSPTSISVALAMVYAGAR
GKSEAELSSALGHTATGLSSRKSILEFYKKILAQQQTDDNVSLMIANAVFVEKNLKVLESYQKELVDTFAAM
FRSVDGGAEKSDMQSEVNEWVKNKTRGKISGFETPADVVMVILNAIYFKGLWETPFKPKNTSPLPFYNKG
SQEIKVETMTRLDNVPFTFEPDFAALELSYKGDRHCMVIVLPREKKGLPKLRDAITVETFKKIQGSLKNDTV
QIQLPKFNLETDYSLVPALTKLGVRSIFSDADLSGITGDRGLRVTEVRHKAAIEVNEEGTVATGVTKVDMGR
RIAGHFIVDRPFLFYIREKETGRVLFLGQVHELPAAKLVLG

>s61c10_g6964.t2_Chr10_Serpin17 XP_002415308.5 (PI=99.8) intracellular coagulation inhibitor 2 [Ixodes scapularis]
MSARLRHPQLGFPARLEKYRSARMERRSAQASRAGDEQPTTVPRETRQTNYLTMTRLLWLFAAITASLA
QDDISQDKWKLARANNYLGLNLLKQLPSNDKTNVFFSPFSVSTAMGMAYAGARGDTLEQLTLNFGYAAD
ELNEGKVLALFKEQLQSTNDLPHDYTLNIANAAVAQEGYGILPEYTDALTSSFGAEYIEADFQKHGQEAIQK
INAWVSNRTHGKVQSLFDEPPDFSTRLILLNAIYYKGTWLYEFDKTKTKPRSFYNGGVEKVQVPMMRLKS
TLNHTYNAILNADLVDLPYVGNDFSMTIILPREKTGLASLKSVLTSQTLNLALQNMYPKDMKLKLPKFKLDT
KYTLKPTLEAMGITKIFSADADLSGISGSRNLYVSDVLHKAVLEVNEEGSEAAAVTGFVIQLRTAAFVTPPPL
PKVYVDHPFIFLIRNSRTNTIMFLGEINAL

>s62c10_g7123.t1_Chr10_Serpin32 XP_002416150.3 (PI=98.6) intracellular coagulation inhibitor 1-like [Ixodes scapularis]
MAILSALTFLQVICMTIGNQVTTVSNNLGFDLLRALPTSKEGNIFFSPYGVSSMVGMLYAGCQGKSNDILFR
QLKFPAFVMTFSDVLGAFAQRAKETGFVDALWPNLQVANAVLIDESFNVSYIYKKTLTRSFSAALLKVNFEK
SGQFLMRYVNAWVQRHTDYQIEGFVDHEFDPETKLVLLNAVYFKGAWNALFSKKNTTKRQFLNDGVTGA
QVDTMSNTFTVNYAASKALSAVVVDLPYRSNNLSMTILLPRENDGVDRLKQSLTGQRFRNLVSDMHAIRA
AVHLPKFRLENKYFLTDPLKSAGINEIFSESVDLSGISTQSNLRVPGVEHKAVVEVNEAGSEASPSTSGLVS
KSVETLKFWVDHPFLFFIRNTVTRDILFMGQVNRL

>s63c11_g165.t1_Chr11_Serpin31-like XP_029832058.2 (PI=97.9) leukocyte elastase inhibitor [Ixodes scapularis]
MEASLSNHILNFSIDLYKQLKPSGKDTAGNVFFSPFSIAAALSMALAGARGNTAEQIAAILHSNDDKIHNHFS
SFLCKLPSYAPDVALHIANRMYSEQTFQPKAEYTTLLQKSYDSTIKAVDFAGNADRVRQEVNAWVEEVTR
SKIRDLLAPGTVDASTSLILVNAIYFKGLWDSQFKPSATQPEDFHLTPQTSKTVDMMHQKGDFKMGHCSD
LKVTALEIPYKGNKMSMIILLPEDVEGLSVLEEHLTALKLSALLGGMYVTPDVNLRLPKFKLEQSIGLKDVLM
AMGVKDFFTSLADLSGISATGDLYASDVIHKAFVEVNEEGTEAAAATAVPMMFGCAIFPEVVNFYVDHPFM
FLIHSHDPDVVLFMGSIRELL

>s64c11_g301.t1_Chr11 XP_040067237.1 (PI=96.4) leukocyte elastase inhibitor [Ixodes scapularis]
MHHHPPIQKHPEHPNMAVNLACPLFDFALDLYKQLLVQTGSTANIFYSPFSIAAALSMTLAGARHNTAKQV
EHVMHLEAGTVHKHFFDVLSMIDSYAPDVTLQVANRLYSDQSFHVLPAYTSLLEEFYKSTMKAVDFKKDP
GASRLEINTWVQEATRSKIKDLLPEGSIDSDTELVIVNAIYFKGLWSFQFNPRATSPKEFHVSKDGTKTVHM
MYKQAKFRMSRCGEHKVSVLEIPYKGKRASMVILLPDKMDGLSDLEKALTSSTFRKILDGLTRETLVDLRL
PRFKLEQTTNLKDTLMAMGIHDLFSDSADLSGMSDNENSEVSAAIHKAFVEVNEEGTEAAAASAFVRIAR
CAVHSVPFTVDHPFLFVIRSHDPDILLFMGSVRQV

>s65c11_g303.t1_Chr11 XP_040067237.1 (PI=96.4) leukocyte elastase inhibitor [Ixodes scapularis]
MHHHPPIQKPPELPNMAANLACPLFDFALDLYKQLLVQTGSTANIIYSPFSIAAALSMTLAGARHNTAKQVE
HVMHLEAGMVHKHFSDVLSMIDSYAPDVTLQVANRLYSNQSFHVLPAYTSLLEEFYKSTMKAVDFKKDPG
ASRLEINTWVQEATRSKIKDLLPEGSIDSDTELVIVNAIYFKGLWSFQFNPRATSPKEFHVSKDGTKPVNM
MYKQAKFRMSRCGEHKVSVLEIPYKGKRASMVILLPDKMDGLSDLEKALTSSTFRKILDGLTGETLVDLRL
PRFKLEQTTNLKDTLMAMGIHDLFSDSADLSGMSDNENSEVSAAIHKAFVEVNEEGTEAAAASAFVRIAR
CAVHGVPFTVDHPFLFVIRSHDPDILLFMGSVRQV

>s66c11_g663.t1_Chr11_Serpin30 XP_040067237.1 (PI=96.4) leukocyte elastase inhibitor [Ixodes scapularis]
MHHHPPIQEHPELPNMASNLACPLFDFTLDLYKQLLVQTGSTANIFYSPFSIAAALSMTLAGARHHTAKQV
EHVMHLEASTVHKHFSDVLSKIDSCAPDVTLQVANRLYSDQSFSVLPTYTSLLEEFYKSTMKAVDFKNDP
GASRLEINAWVEEATRSKIKDLLPEGSIDSDTVLVIVNAIYFKGLWSFQFNPRATSPQEFHVSKDGTKTVDM
MYKQAKFRMSRCDEYKVSVLEIPYKGKRASMVILLPDEMDGLSDLEKALTSSTFRKILDGLTRETLVDLRL
PRFKLEQTTNLKDTLVAMGIHDLFSDSADLSGMNSNESLKVSAAIHKAFVEVNEEGTEAAAASAFAVNARC
AVYGVPFSVDHPFLFVIRSHDPDIILFMGSVRQV

>s67c11_g1688.t1_Chr11_Serpin28-like CAN8031797.1 (PI=97.3) unnamed protein product [Ixodes persulcatus]
MASDFGDSLLSFSVDIYKKLKNEGSGAGNIICSPFSIAAALSMTLAGARHDTAKQVSSALHVQHDTIHGHF
ADFLSKLPAYAPDVILHVANRLYSEQTYKTLEEFTRLLEKSYGTTIENVDFRRNFDQARLQVNAWVEEATR
SKIKDLLAKGTVDASTSLIIVNAVYFKGLWHDQFDPMRTSQQEFHETTDRSKMVDMMYQKKRFRMSRHP
DVKVSALEIPYKGKKTSMVILLPEEVDGLAGLEEALTASNLTEILQGLSHQGDIELTLPKFKLEQAVGLKKVL
ATMGVEDLFDPSKCDLSGISADKDLVVSDVIHKAFVEVNEEGTEAAAATAVMMVKYCLQFPTRFTVDHPFL
FLIRSHDPDVVLFLGSVRQ

>s68c11_g4528.t1_Chr11 XP_042144458.1 (PI=100) intracellular coagulation inhibitor 3-like [Ixodes scapularis]
MKLARAQNHFSLKLLKELSSEAPESNLFFSPTSISVALAMVYAGARGKSEAELSTVLGHTAAGLPSRESILE
SYKRILAKQQTDDNVSLMIANAVFVMKTLKVLESYRKELVDIFAALFRSIGVGAENSDMESEVNEWVKNKT
RGKISGFKIPPNTIMAILNAIYFKGLWNTSFDPNNTSPLPFHNKGSKVVLVKTMTRVSKVPFASEPDFEAIEL
SYKGDEHCMVIILPRQRNGLSKLRDAMTVETIEKIQGSLKEKTVKIQLPKFDLKTKYGLVPALKKLGVRSIFS
DADLSGITGNRGLVVTDVLHKAAIQVNEEGTVAAAATVIIFKSALQPFVVNRPFLFYIREKATGRMLFLGEV
HELPAPKPTLG

>s69c13_g215.t1_Chr13 XP_040075471.1 (PI=100) intracellular coagulation inhibitor 3-like [Ixodes scapularis]
MMLAEKVFLVLFVLLAGDSECYAEDSDIKLAQAQNHFALKLLKELSSEAPESNIFFSPTSIFVALAMVYAGA
RGKSETELSSALGHTSAGLSSRESILESYKKILAQQQTDDNVSLMIANAVFVEKNLKVLESYQKELVDTFAA
MFRSVDGGAEKSDMESEVNEWVKNKTRGKILGFKIPADVVMVILNAIYFKGLWETPFNPNNTSPLPFYNK
GSQEIEVETMTRLDNVPFTFEPEFAALELSYKGDRHCMVIVLPHEKKGLPKLRDAITVETIKKIQGSLKNDT
VKIQLPKFNLETDYSLVPALTKLGVRSIFSDADLSGIAGDRGLRVTEVRHKAAIEVNEEGTVATGITIVEIGRS
LPRPFVVDRPFLFYIREKATGRVLFLGEVHELPAVKPALG

>s70J234_g1431.t1_UnplacedContig XP_040067806.1 (PI=98.4) intracellular coagulation inhibitor 2-like [Ixodes scapularis]
MMLAQNVLIVLFVLLTGDGECNAEDSDMKLARAQNHFALKLLKELCSEEPESNIFFSPTSISVALAMVYAVA
RGKSEAEFSTALGHTAAGLPSRESTLESYKKILAEQQTDDNLSLMIANAVFVQKKLKVLKSYRKELVDIFAA
MYRSVDVGGGKSAMESEVNQWVKNKTKDQISGFKIPAGTITALLNAINFKGLWETPSDLKYSFSLPFYNK
GSEVVMAETMSRDGTVLFTSEPGLKCEAIEYPYKGGRHSMIFVFPKEKNGLAELRQAITVESIEKIQRNLK
ETVLMFQLPKFDLKTEYNLVPALKKLGVGSIFSDADLSGIPGGEGQVVTEVQHKAAIEINEEGTVAAAATAV
RASKNRTQSFVVNRPFLFYICEKATGRLLFLGEVHALPAIK
